# Chromatin accessibility is associated with the changed expression of miRNAs that target members of the Hippo pathway during myoblast differentiation

**DOI:** 10.1038/s41419-020-2341-3

**Published:** 2020-02-24

**Authors:** Huanhuan Zhou, Yue Xiang, Mingyang Hu, Yueyuan Xu, Ye Hou, Xiaolong Qi, Liangliang Fu, Yu Luan, Zhangxu Wang, Xinyun Li, Yunxia Zhao, Shuhong Zhao

**Affiliations:** Key Laboratory of Agricultural Animal Genetics, Breeding, and Reproduction of the Ministry of Education and Key Laboratory of Swine Genetics and Breeding of Ministry of Agriculture, Huazhong Agricultural University, 430070 Wuhan, China

**Keywords:** Chromatin, miRNAs

## Abstract

miRNAs reportedly participate in various biological processes, such as skeletal muscle proliferation and differentiation. However, the regulation of differentially expressed (DE) miRNAs and their function in myogenesis remain unclear. Herein, miRNA expression profiles and regulation during C2C12 differentiation were analyzed in relation to chromatin states by RNA-seq, ATAC-seq, and ChIP-seq. We identified 19 known and nine novel differentially expressed miRNAs at days 0, 1, 2, and 4. The expression of the differentially expressed miRNAs was related to the chromatin states of the 113 surrounding open chromatin regions defined by ATAC-seq peaks. Of these open chromatin regions, 44.25% were colocalized with MyoD/MyoG binding sites. The remainder of the above open chromatin regions were enriched with motifs of the myoblast-expressed AP-1 family, Ctcf, and Bach2 transcription factors (TFs). Additionally, the target genes of the above differentially expressed miRNAs were enriched primarily in muscle growth and development pathways, especially the Hippo signaling pathway. Moreover, via combining a loss-of-function assay with Q-PCR, western blotting, and immunofluorescence, we confirmed that the Hippo signaling pathway was responsible for C2C12 myoblast differentiation. Thus, our results showed that these differentially expressed miRNAs were regulated by chromatin states and affected muscle differentiation through the Hippo signaling pathway. Our findings provide new insights into the function of these differentially expressed miRNAs and the regulation of their expression during myoblast differentiation.

## Introduction

Skeletal muscles account for ~40% of the body weight of humans^1^ and play important roles in motor movements and energy metabolism^2^. Myogenesis is a multistep process that includes myoblast proliferation, myoblast differentiation into postmitotic myocytes, and finally the fusion of myocytes into multinucleated myotubes^3,4^. The transcription factors (TFs) MyoD, Myf5, MyoG, MRF4, and MEF2 coordinate the expression of muscle-specific genes^5^, driving myoblasts to exit the cell cycle to undergo myogenesis. Accumulating evidence has shown that muscle development is regulated by microRNAs (miRNAs) in addition to the regulators described above. miRNAs have been reported to be involved in many biological processes, including skeletal myogenesis^6,7^ and adipogenesis^8^. Muscle-specific miRNAs, including miR-1^9^, miR-133^10^, miR-206^11^, miR-208^12^, and miR-499^13^, have been considered key regulators with different targets during muscle development. Moreover, ubiquitously expressed miRNAs, such as miR-128^14^, miR-27^15^, miR-155^16^, miR-378^17^, miR-486^18^, miR-29^7^, miR-29b^19^, miR-34b^20^, miR-152^21^, and miR-660^22^, have been found to be crucial factors affecting the formation of skeletal muscle.

As important regulators of gene expression, TFs regulate target genes, including miRNA genes, mainly through binding to their promoter regions surrounding transcription start sites or enhancer elements^23,24^. The assay for transposase-accessible chromatin with high-throughput sequencing (ATAC-seq) method can map chromatin accessibility at the genome level^25^. This method can also predict TF occupancies. Additionally, previous chromatin immunoprecipitation followed by sequencing (ChIP-seq) analyses showed that MyoD and MyoG can bind to upstream regions of miR-1, miR-133, and miR-206 primary miRNAs (pri-miRNAs) and are thus likely to regulate the enhanced expression of these miRNAs during myogenesis^26^. Moreover, MyoD, MEF2, and SRF can specifically bind their respective sites in the upstream enhancer regions of mir-1-1 and mir-1-2 to directly regulate miR-1 expression^27^, and the SRF expression was repressed by miR-133 in turn^9^. Moreover, MyoD can cooperate with MEF2 to activate the transcription of the pri-miRNAs encoding miR-1-2 and miR-133a-1 via an intragenic enhancer between the mir-1-2 and mir-133a-1 coding regions in skeletal muscle^28^. However, the function of chromatin states and their associated TFs in the regulation of miRNA expression was still a missing part and is also the time course study of miRNA expression in myoblast differentiation.

In this study, we identified differentially expressed (DE) miRNAs during C2C12 myoblast differentiation and analyzed their potential target genes and signaling pathways through bioinformatic analysis and experimental verification. We confirmed that the Hippo signaling pathway was responsible for the myogenic differentiation of C2C12 myoblasts and revealed the regulation of miRNA expression by changes in chromatin states by combining genome-wide high-resolution maps of ATAC-seq and ChIP-seq data.

## Materials and methods

### Cell culture

Mouse C2C12 myoblasts were cultured in growth media containing high-glucose Dulbecco’s modified Eagle’s medium (DMEM; HyClone, USA) supplemented with 10% fetal bovine serum (Gibco, USA) at 37 °C under 5% CO_2_ in a humidified incubator. To induce differentiation, the culture medium was replaced with DMEM supplemented with 3% horse serum (Gibco, USA) for 4 days.

### Small RNA library preparation and sequencing

Total RNA was extracted from cells at days 0, 1, 2, and 4 by using TRIzol reagent (Invitrogen, USA). Triplicate samples were established. The RNA concentration was quantified using a NanoDrop 2000 spectrophotometer (Thermo, USA). Then, the triplicate samples were pooled for small RNA library preparation. Size fractionation of total RNA was performed to select fragments in the range of 18–30 nucleotides. The 3′ adaptors were ligated with the fragments by using truncated T4-RNA ligase, and the 5′ adaptors were similarly ligated using T4-RNA ligase. cDNA was synthesized by reverse transcription and used for polymerase chain reaction (PCR) amplification. Then, the PCR products were purified to construct the libraries. After quality control, four libraries were sequenced in an Illumina HiSeq high-throughput sequencing instrument.

### RNA sequencing (RNA-seq) analysis

After removing low-quality tags and several kinds of contaminants from the raw sequencing reads, the clean datasets were processed with mirDeep^29^ (v2.0.0.5), annotated to the miRBase database^30^ (version 20) as the miRNA reference and aligned to the mouse reference genome (mm10) to predict novel miRNAs. Differentially expressed miRNAs between the four analyzed time points were identified using DESeq2^31^ as those with |log_2_FoldChange| (|log_2_FC|) ≥ 1 and *p* < 0.05.

### miRNA target prediction and network construction

The target genes of known miRNAs were predicted by the online miRNA target prediction tools TargetScan (http://www.targetscan.org/) and miRDB (http://mirdb.org/). Moreover, the target genes of novel miRNAs were predicted by IntaRNA 2.0 (https://github.com/BackofenLab/IntaRNA/) and DIANA (http://diana.imis.athena-innovation.gr/DianaTools/index.php). Then, the overlapping genes were selected as the final set of miRNA target genes. The networks between miRNAs and their target genes were constructed using Cytoscape software^32^.

### Gene ontology and kyoto encyclopedia of genes and genomes analyses

All predicted target genes were subjected to gene ontology (GO) term analysis and Kyoto Encyclopedia of Genes and Genomes (KEGG) pathway analysis via DAVID Bioinformatics Resources 6.8 (https://david.ncifcrf.gov/). A *p* value of less than 0.05 was considered to indicate a significant difference.

### Real-time quantitative PCR analysis

Triplicates of total RNA isolated from each sample at days 0, 1, 2, and 4 were subjected to residual DNA removal via DNase I (Thermo, USA). Samples were reverse transcribed to cDNA with miRNA-specific primers or random primers and a RevertAid First Strand cDNA Synthesis Kit (Thermo, USA) according to the manufacturer’s instructions. Quantitative PCR (Q-PCR) was performed with SYBR Green PCR Master Mix (Toyobo, Japan) in 384-well plates in a CFX384 Real-Time PCR Detection System (Bio-Rad, USA). The reactions were incubated at 95 °C for 10 min and subsequently subjected to 40 PCR amplification cycles at 95 °C for 30 s, 60 °C for 30 s, and 72 °C for 20 s. All reactions were run in triplicate. The expression fold changes were calculated using the 2^−∆∆Ct^ method^33^ with U6 and GAPDH as the internal controls as appropriate. All primers used for cDNA synthesis and Q-PCR are listed in Tables S4 and S5.

### Luciferase activity assay

The 3′ untranslated region (UTR) fragments containing the predicted binding sites or corresponding mutated binding sites of potential target genes were amplified from total cDNA of C2C12 myoblasts or synthesized by TsingKe (TsingKe, P.R.C.). The fragments were then inserted into the psiCHECK-2 vector (Promega, USA) by using the restriction enzymes XhoI and NotI. The constructs were cotransfected with the miRNA mimic or scrambled negative control (RIBOBIO, P.R.C.) into BHK-21 cells by using Lipofectamine 2000 (Invitrogen, USA). All transfections were conducted in at least triplicate in accordance with the manufacturer’s recommendations. The relative luciferase activity was analyzed by the Dual-Luciferase Reporter Assay System (Promega, USA) in a luminometer (PE EnSpire, USA) after a 24 h incubation in 96-well plates. All primers and sequences used in the luciferase activity assay are shown in Table S6.

### Binding profiles of ChIP-seq and ATAC-seq analysis

The ATAC-seq datasets used were from our unpublished study. The downloaded ChIP-seq datasets for MyoD and MyoG were from a previous study^34^. The potential regulation of miRNAs by TFs was examined by analyzing the normalized signals from genomic regions of approximately ±10 kb surrounding the pri-miRNAs of differentially expressed miRNAs with the multiBigwigSummary package of deepTools (https://deeptools.readthedocs.io/en/latest/index.html). Motif enrichment analysis of open chromatin regions with or without colocalization of MyoD or MyoG binding was analyzed with HOMER (v4.10, http://homer.ucsd.edu/homer/).

### Western blotting

Total protein was extracted from cells with RIPA lysis buffer (Sigma, USA) supplemented with 1% protease inhibitor and 1% phosphatase inhibitor, separated by SDS-PAGE, and transferred to polyvinylidene fluoride membranes (Millipore, USA). Membranes were blocked with 5% nonfat powdered milk at room temperature for 2 h and were then incubated at 4 °C overnight with primary antibodies specific for the following proteins: MyoD (1:1000, ABclonal, P.R.C., A0671), MyoG (1:500, Abcam, USA, ab1835), MyHC (1:750, Sigma, USA, M4276), and β-tubulin (1:1000, Sungene, P.R.C., KM9003T). Then, membranes were incubated with HRP-labeled goat anti-rabbit IgG (H + L) or anti-mouse IgG (H + L) secondary antibodies (Beyotime, P.R.C.). Protein signals detected with Immobilon Western Chemiluminescent HRP Substrate (Millipore) were visualized in an ImageQuant LAS4000 mini instrument (GE Healthcare Bio-Science, USA).

### Immunofluorescence

C2C12 cells cultured in 12-well plates were washed twice with phosphate-buffered saline (PBS) and fixed with ice-cold 4% paraformaldehyde for 15 min. Then, the cells were again washed twice with PBS and incubated in ice-cold 0.3% Triton X-100 at room temperature for 10 min. The cells were then washed three times and incubated in blocking solution (3% bovine serum albumin, 10% fetal bovine serum and 0.3% Triton X-100) at room temperature for 2 h. Next, the cells were again washed three times and incubated at 4 °C overnight with primary antibodies specific for the following proteins: MyoD (1:100, Santa Cruz, USA, sc-32758×), MyoG (1:100, Abcam, USA, ab1835), and MyHC (1:200, Sigma, USA, M4276). The cells were then washed three times with PBS and incubated with anti-mouse IgG (H + L), F (ab′) 2 Fragment (Alexa Fluor® 555 Conjugate, CST, USA, 4409) for 2 h at room temperature in the dark. Finally, the cells were washed three times with PBS and stained with 4′,6-diamidino-2-phenylindole (DAPI) in the dark. Images were acquired using an OLYMPUS IX73 TH4-200 system (OLYMPUS, Japan).

### RNAi assay

For the *Yap1* RNAi assay, when the cells were ~40% confluent, C2C12 myoblasts were transfected with siRNA (RIBOBIO, P.R.C.) using Lipofectamine 2000 (Invitrogen, USA) according to the manufacturer’s instructions. After 24 h, the medium was replaced with DMEM supplemented with 3% horse serum (Gibco) for 4 days.

### Generation of knockdown cells using CRISPR/Cas9 genome editing

A lentiviral gene transfer system was applied to inhibit the expression of *Wwtr1*. The 20 nucleotide guide sequences were obtained from a previous study^35^ and synthesized by TsingKe (TsingKe, P.R.C.). Single guide RNA was cloned into the lentiviral vector lentiCRISPRv2 (Addgene plasmid #52961), and 293T cells were cotransfected with the VSVG, REV, and MDL plasmids for 48 h using Lipofectamine 2000 (Invitrogen, USA) according to the manufacturer’s recommendations. The viral supernatant was collected and filtered through a 0.45 μm membrane for the infection of C2C12 myoblasts aided by polybrene. At 48 h post infection, knockdown cells were selected with puromycin (Gibco, USA) until all normal control cells died. Then, these cells were expanded and screened by genomic sequencing and further functional assays. The guide RNA sequence for *Wwtr1* used in the study was as follows: GCAGTGTCCCAGCCGAATCT.

### Statistical analysis

All data are presented as the means ± SDs. Student’s *t*-test was used to analyze statistical significance. Values of *p* < 0.05 were considered statistically significant.

## Results

### Differentially expressed miRNAs during C2C12 myoblast differentiation

**Fig. 1:**
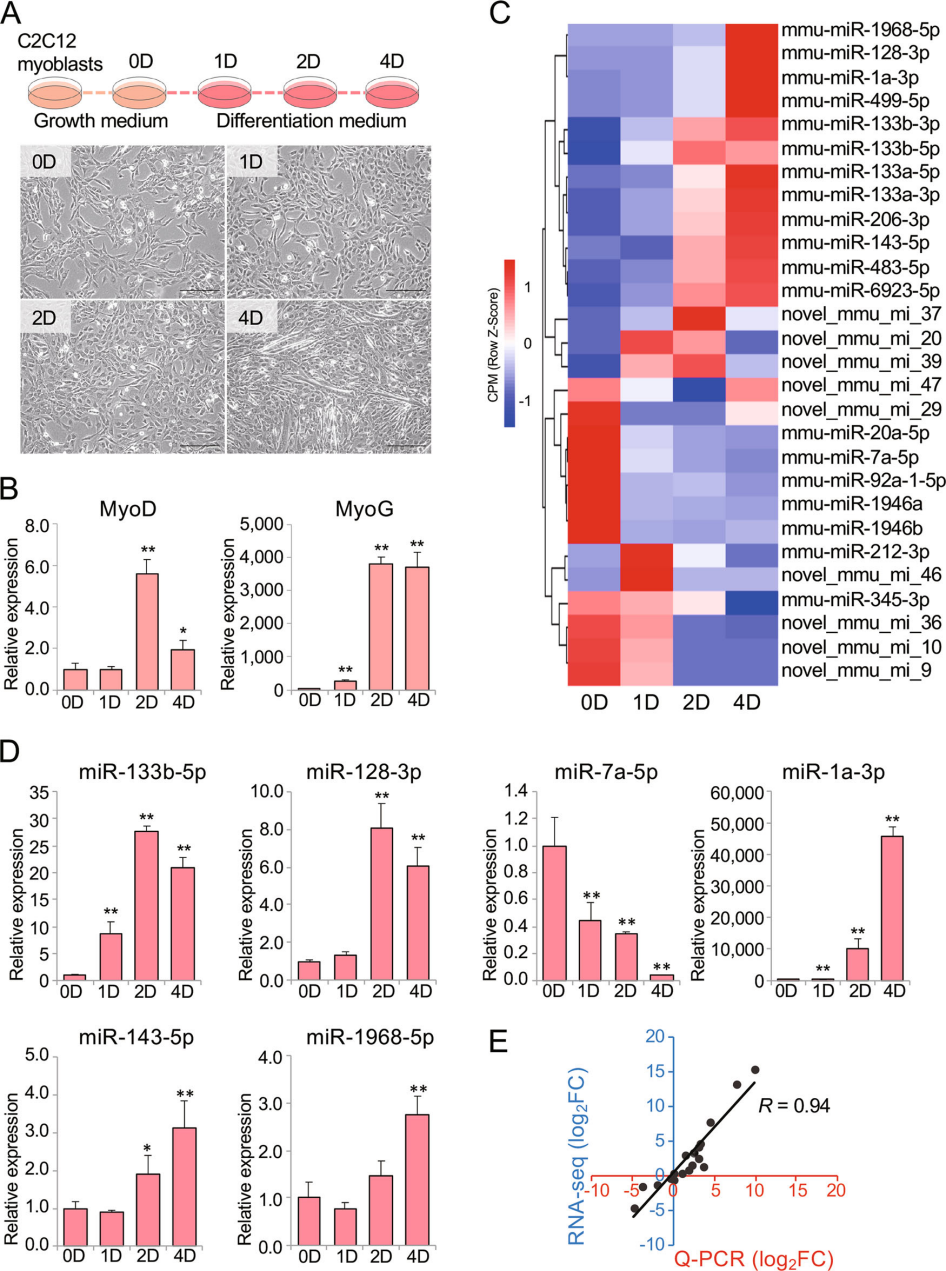
Differential expression of miRNAs during C2C12 myoblast differentiation. **a** Schematic for stimulating the differentiation of C2C12 myoblasts into myotubes and morphological images of C2C12 myoblasts cultured in GM or in DM for 0, 1, 2, and 4 days (×100). **b** Expression of *MyoD* and *MyoG* during cell differentiation as determined by Q-PCR. The expression fold change is relative to day 0 in GM culture. **c** Hierarchical clustering of differentially expressed miRNAs on days 0, 1, 2, and 4 during C2C12 differentiation. **d** Expression of miRNAs on days 0, 1, 2, and 4 of cell differentiation via stem-loop Q-PCR. The expression fold change is relative to day 0 in GM culture. **e** Correlation analysis of stem-loop Q-PCR and miRNA sequencing data. The *x*-axis and *y*-axis show the values obtained by Q-PCR and RNA-seq, respectively. *GM*, growth medium; *DM*, differentiation medium. The error bars indicate the standard errors of the mean from three independent experiments. *p < 0.05; **p < 0.01.

To study the regulatory role of miRNAs in myogenesis, small RNA libraries were prepared from the time course of C2C12 myoblast differentiation, including in proliferating myoblasts (day 0) as well as 1, 2, and 4 days after changing the medium to differentiation medium. During this time course, the myoblasts gradually developed into myotubes (Fig. 1a). In addition, both the mRNA and protein expression of *MyoD* and *MyoG* were significantly upregulated during C2C12 myoblast differentiation (Figs. 1b and S1). These results indicated that C2C12 myoblasts successfully differentiated into myotubes in vitro. After the removal of low-quality tags, adaptor trimming and other contaminants from the sequencing data, 13.8–15.4 million clean reads per library were obtained. Here, 96.96–99.08% of the clean reads could be aligned to the mm10 mouse reference genome, of which the known miRNAs accounted for 89.99–97.45%. Moreover, novel miRNAs that were not yet identified accounted for 0.02–0.04% (Table 1).

**Table 1:** Numbers of miRNA reads of C2C12 myoblast differentiation on days 0, 1, 2, and 4.

Time points	Total clean reads	Mapped to genome reads	Known miRNA reads	Novel miRNA reads
Day 0	15,375,673	14,907,530 (96.96%)	13,836,657 (89.99%)	5416 (0.04%)
Day 1	14,455,343	14,112,887 (97.63%)	13,352,334 (92.37%)	6365 (0.04%)
Day 2	13,782,068	13,559,049 (98.38%)	13,106,104 (95.10%)	2853 (0.02%)
Day 4	14,106,896	13,977,697 (99.08%)	13,747,568 (97.45%)	3492 (0.02%)

A total of 28 significantly differentially expressed miRNAs (|log_2_FC|) ≥ 1 and *p* < 0.05) were identified. Moreover, these miRNAs could be extended to 33 differentially expressed miRNAs, namely, 24 known miRNAs and nine novel miRNAs, with reference to their respective precursor source (Table S1). The cluster analysis revealed that 13 miRNAs were in one group that was downregulated overall, whereas 15 miRNAs clustered into another group that was upregulated overall (Fig. 1c). Furthermore, six random miRNAs (miR-133b-5p, miR-128-3p, miR-7a-5p, miR-1a-3p, miR-143-5p, miR-1968-5p) from this set were selected for stem-loop Q-PCR (Fig. 1d). Their expression patterns also displayed a high degree of consistency with the miRNA sequencing data (*R* = 0.94, *p* < 0.01, Fig. 1e).

### Changes in open chromatin and transcription factor binding altered miRNA expression during myoblast differentiation

**Fig. 2:**
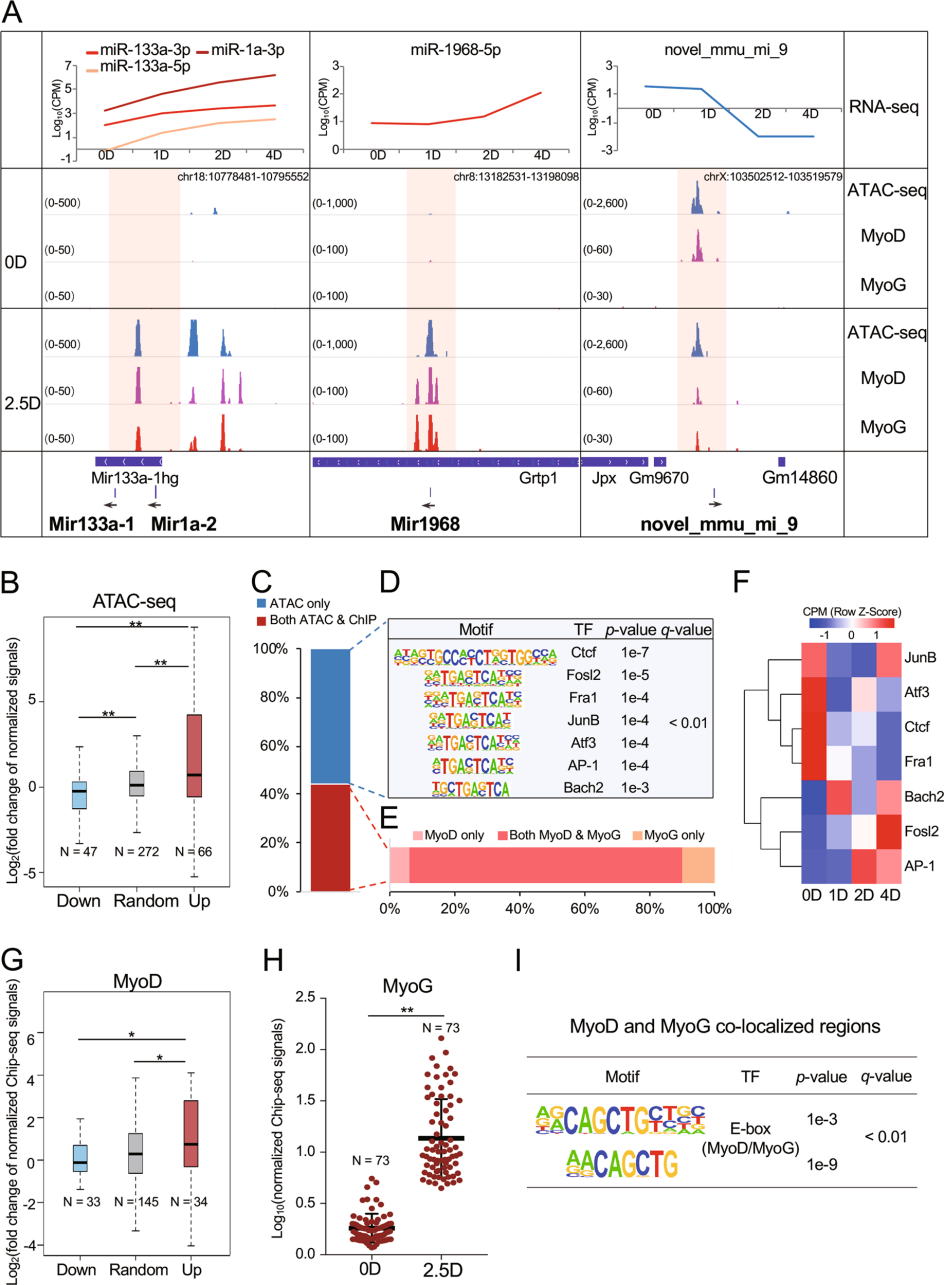
Changes in chromatin states around differentially expressed miRNAs between C2C12 myoblasts and myotubes. **a** Integrative genomics viewer visualization of intensities of ATAC-seq and ChIP-seq surrounding the mir133a-1, mir1a-2, mir1968, and novel_mmu_mi_9 gene loci and the expression patterns of these miRNAs. **b** Boxplots showing the fold change patterns of normalized ATAC-seq intensities. **c** Statistics of colocalization of ATAC-seq-defined open chromatin regions and TF binding. **d** Motif enrichment analysis of open chromatin regions without colocalization of MyoD or MyoG binding. **e** Statistics of open chromatin region colocalization with MyoD or MyoG binding. **f** Expression of TFs enriched in (D) by RNA-seq. **g** Boxplot of ChIP-seq intensities of MyoD in the genomic region ~±10 kb around the pri-miRNA loci of differentially expressed miRNAs and random non-differentially expressed miRNAs in C2C12 myoblasts cultured in GM (0D) and then in DM for 60 h (2.5D). **h** Scatter plot showing normalized ChIP-seq intensities of MyoG binding surrounding the ~±10 kb genomic region around the pri-miRNA loci of differentially expressed miRNAs in C2C12 myoblasts cultured in GM (0D) and then in DM for 60 h (2.5D). **i** Motif enrichment in MyoD and MyoG-colocalized regions. *p < 0.05; **p < 0.01.

To explore the regulation of the 33 differentially expressed miRNAs identified in our study, we generated genome-wide high-resolution maps of ATAC-seq data and combined them with published ChIP-seq data to investigate the chromatin states and TF occupancies surrounding the corresponding 30 pri-miRNA loci. By using Integrative Genomics Viewer to visualize the binding profiles from ATAC-seq and ChIP-seq data surrounding these pri-miRNA loci, we found that the changes in the ATAC-seq signals around the differentially expressed miRNAs were consistent with the differences in the miRNA expression levels between day 0 and day 2.5 during C2C12 myoblast differentiation (Fig. 2a). Specifically, highly overlapping occupancies of MyoD and MyoG surrounding these pri-miRNAs were observed in C2C12 cells cultured in differentiation medium for 2.5 days (Fig. 2a), suggesting that these two factors contribute to the regulation of these miRNAs.

We further separated these pri-miRNAs into two groups according to the expression patterns of the miRNAs that they transcribed and randomly selected triplicate data for 33 non-differentially expressed miRNAs from the miRBase database as a reference. As expected, a significantly strong signal of ATAC-seq in the group of upregulated differentially expressed miRNAs and a significantly weak signal of ATAC-seq in the group of downregulated differentially expressed miRNAs were observed (Fig. 2b). This result confirmed that these miRNAs were indeed regulated by the states of chromatin structures that directly affected the active or inactive gene regulatory regions. However, the specific TFs binding in the above 113 open chromatin regions defined by ATAC-seq peak loci were unclear. Here, occupancy by the TFs MyoD and MyoG^34^, which control myoblast differentiation, were observed around the miRNAs and were colocalized with the ATAC-seq signals (Fig. 2a). Further investigation showed that 44.25% of the ATAC-seq peaks around the miRNA sequences were occupied by MyoD or MyoG (Fig. 2c). The motif enrichment and gene expression analyses showed that seven other unique TFs, namely, the AP-1 family TFs (JunB, AP-1, Atf3, Fosl2, and Fra1), Ctcf, and Bach2 (Fig. 2d), exhibited a strong probability (*q* < 0.01) of binding in the open chromatin regions around these miRNAs. Moreover, the expression profiles of these TFs were indeed changed during myoblast differentiation (Fig. 2f).

Furthermore, we analyzed the changes in MyoD and MyoG intensities around the differentially expressed miRNAs. The changes in MyoD intensities were similar to the changes identified by ATAC-seq (Fig. 2g), showing that MyoD is likely one of the regulatory factors affecting these miRNA gene regulatory regions due to changes in chromatin states. Moreover, the intensities of MyoG were increased on day 2.5 compared with day 0 (Fig. 2h), implying that the increase in MyoG binding in myotubes might bilaterally regulate the differentially expressed miRNAs. Furthermore, the majority of the MyoD and MyoG binding sites around these miRNAs were colocalized in myotubes (Fig. 2a, e). Moreover, their binding motifs were significantly enriched in the regions with colocalization of these two TFs (Fig. 2i). These results indicated that the functions of MyoD and MyoG in regulating differential miRNA expression during myoblast differentiation were reciprocally dependent.

### The target genes of differentially expressed miRNAs were primarily involved in skeletal muscle differentiation pathways

To clearly understand the biological functions of these identified differentially expressed miRNAs, potential targets of known miRNAs were predicted by TargetScan and miRDB, and novel miRNAs were predicted by IntaRNA 2.0 and DIANA. Then, the overlapping targets were subjected to further analysis, and 40–510 target genes of each known miRNA (Table S2) and 5–118 target genes of each novel miRNA (Table S3) were returned. In total, 4485 significant target genes, including 3178 unique genes, were returned.

**Fig. 3:**
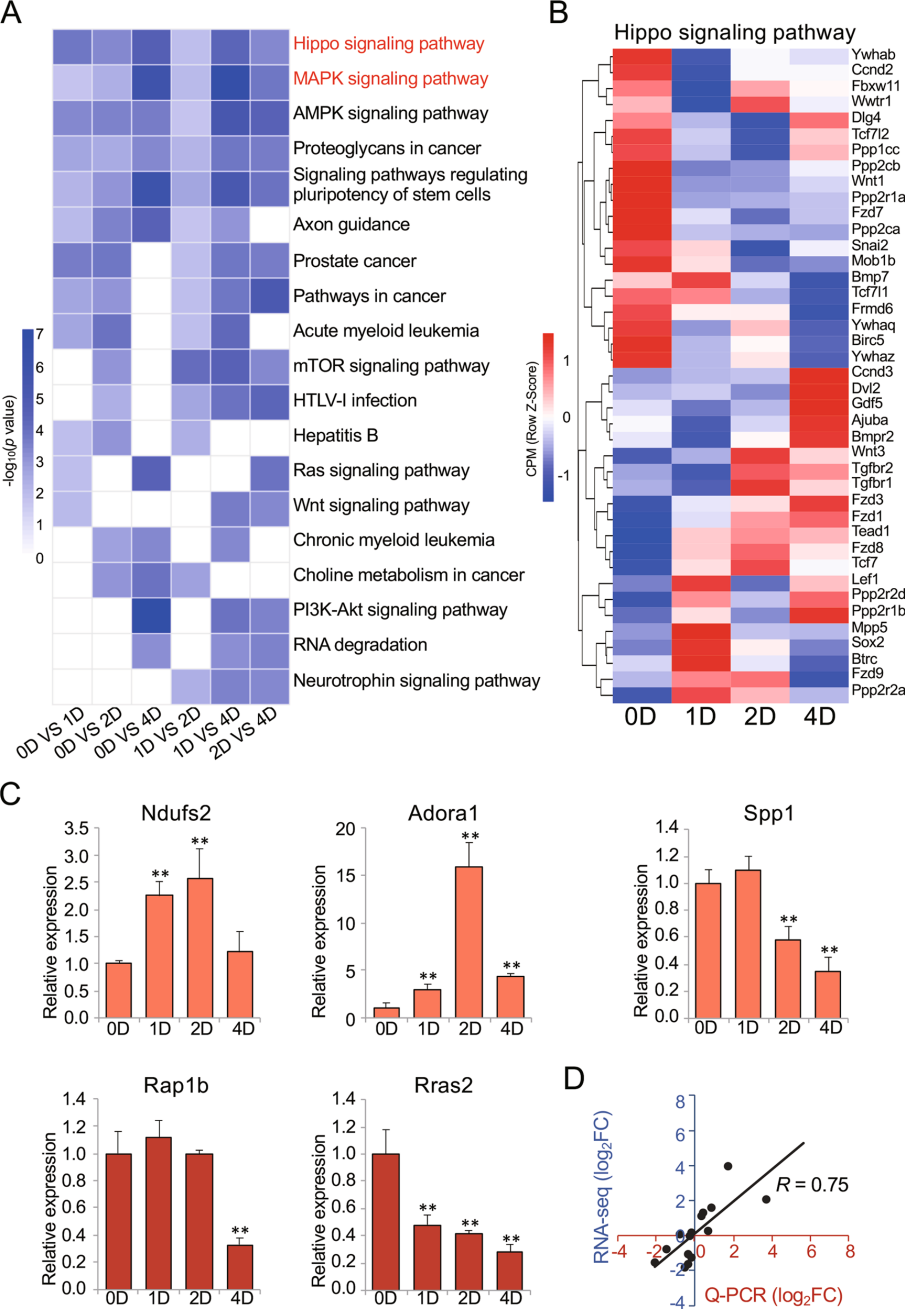
KEGG pathway annotation and RNA-seq dataset analysis. **a** KEGG pathways most significantly enriched in the top 20 differentially expressed miRNA targets ranked by the enrichment score (−log_10_(*p* value)). **b** Heat map and clustering patterns of miRNA targets in the Hippo signaling pathway during C2C12 myoblast differentiation as determined by rRNA-depleted RNA-seq data. **c** Expression of genes as quantified by Q-PCR. The expression fold change is relative to day 0 in GM culture. **d** Scatter plot showing the two general methodologies. The *x*-axis shows the values obtained by Q-PCR, and the *y*-axis shows the values obtained by RNA-seq. *p < 0.05; **p < 0.01.

Functional annotation via the DAVID database was performed to further investigate the potential biological roles of these target genes. GO analysis indicated that the targets of these miRNAs were involved primarily in a broad range of biological functions described by terms such as regulation of transcription, and DNA-templated; regulation of transcription from RNA polymerase II promoter; and cell migration (Fig. S2). Interestingly, the KEGG pathways were primarily associated with muscle growth and development and included the Hippo, MAPK, Wnt, and cAMP signaling pathways (Figs. 3a and S3). In addition, the cAMP signaling pathway contributes to the MAPK pathway, and Wnt signaling pathways participate directly in the Hippo signaling pathway, as referred to by the KEGG database and other studies on skeletal muscle^36^.

We further examined the expression patterns of these pathways by using the same time course rRNA-depleted RNA-seq data of C2C12 myoblast differentiation (data from our unpublished study) and found that the levels of target genes involved in the Hippo and MAPK signaling pathways indeed changed during C2C12 myoblast differentiation (Figs. 3b and S4). Moreover, five random genes, including two target genes of differentially expressed miRNAs (*Rap1b* and *Rras2*) from the RNA-seq data, were selected for Q-PCR. These results showed generally high consistency between the two datasets (*R* = 0.75, *p* < 0.01; Fig. 3c, d).

### The Hippo signaling pathway was regulated by differentially expressed miRNAs during myoblast differentiation

**Fig. 4:**
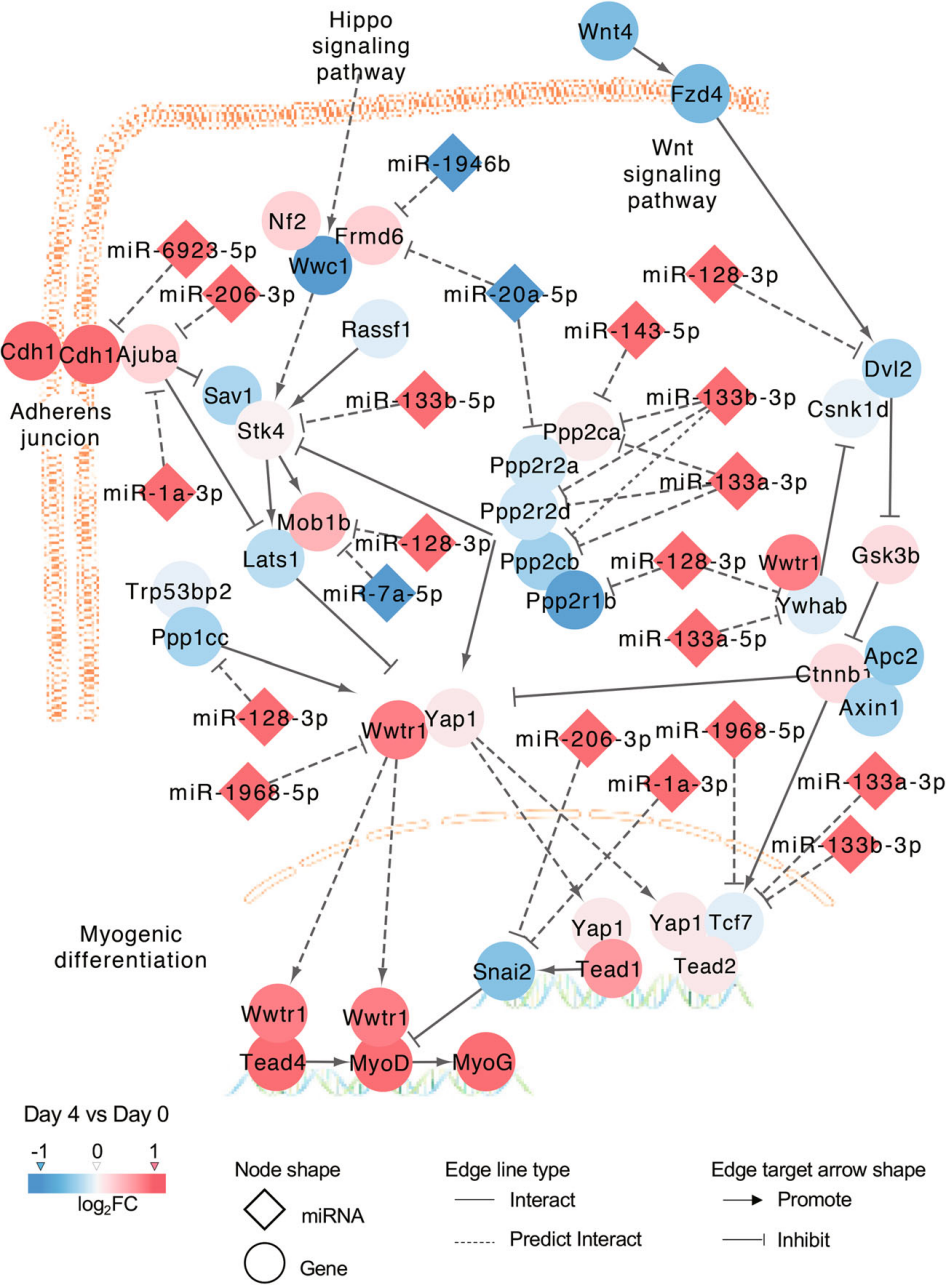
Differentially expressed miRNAs involved in the Hippo signaling pathway and their target genes. Orange red and light red indicate upregulation on day 4 compared with day 0 (orange red, log_2_FC > 1; light red, 0 < log_2_FC < 1), whereas blue and light blue indicate downregulation over this same period (blue, log_2_FC < −1; light blue, −1 < log_2_FC < 0).

**Fig. 5:**
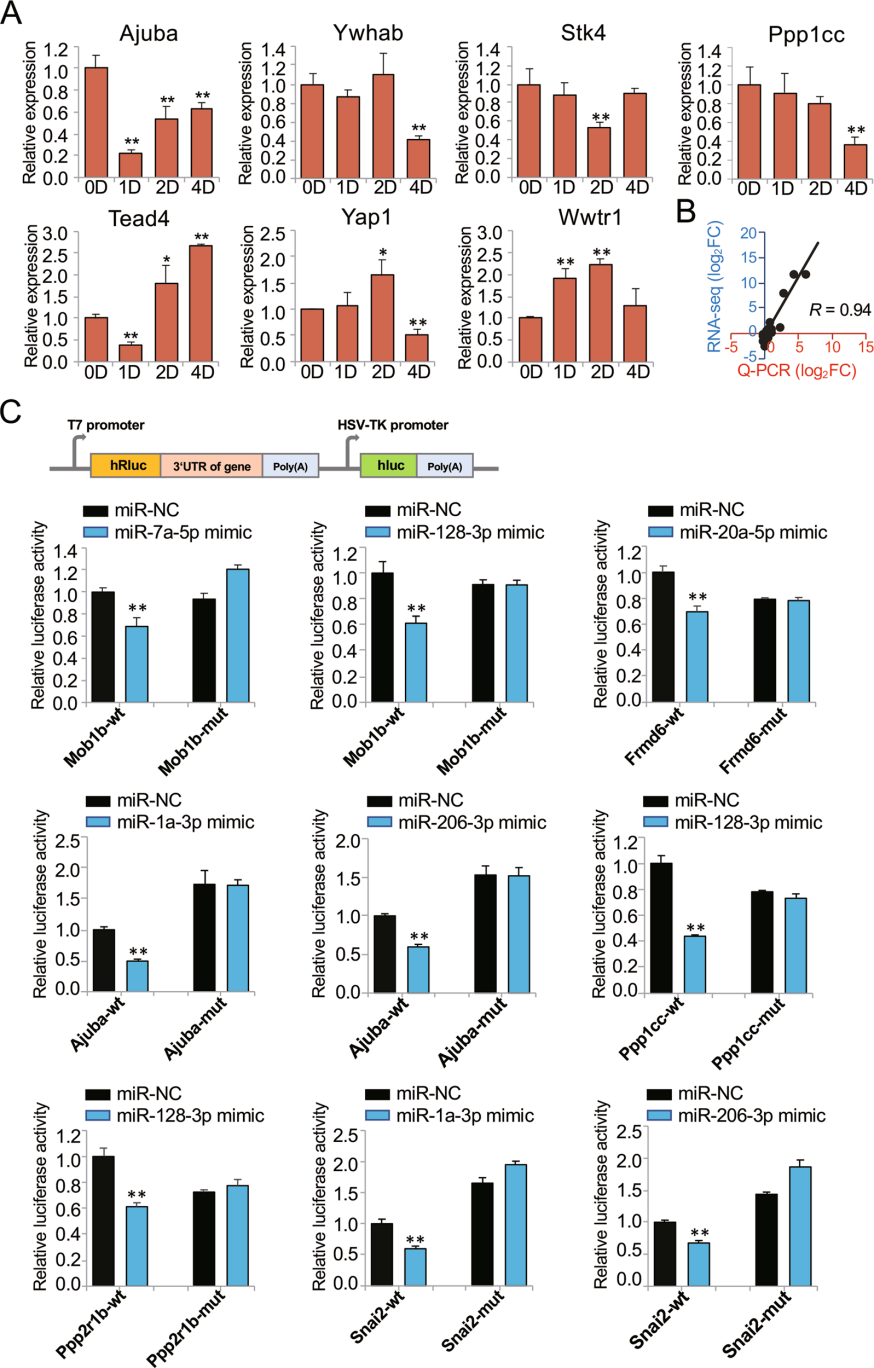
Validation of gene expression levels and targets of miRNAs in the Hippo signaling pathway network. **a** Relative expression levels of genes involved in the network as determined by using Q-PCR. The expression fold change is relative to day 0 in GM culture. **b** Scatter plot showing the general RNA-seq and Q-PCR results. The *x*-axis shows the values obtained by Q-PCR, and the y-axis shows the values obtained by RNA-seq. **c** Validation of interactions between miRNAs and their putative target genes. The relative luciferase activities were measured after cotransfection of BHK-21 cells with psiCHECK-2 constructs that contained putative binding sites or the corresponding mutated binding sites in the 3′-UTRs of potential target genes and either miRNA mimics or negative control (NC) for 24 h. *p < 0.05; **p < 0.01.

The Hippo signaling pathway was an important enriched pathway that involved 13 differentially expressed miRNAs in our study. As visualized in Cytoscape software, 10 Hippo signaling pathway-involved miRNAs (miR-128-3p, miR-1968-5p, miR-1a-3p, miR-133b-5p, miR-133a-5p, miR-206-3p, miR-133a-3p, miR-133b-3p, miR-143-5p, and miR-6923-5p) were upregulated; these miRNAs were likely to be positively regulated by their looser chromatin states during C2C12 differentiation. As expected, most of the target genes in this pathway showed a trend toward downregulation or slight upregulation (Figs. 4, S5, and S6). We further validated the expression of nine Hippo signaling pathway-involved genes, including four target genes of these miRNAs (*Ajuba*, *Ywhab*, *Stk4*, and *Ppp1cc*) and three important genes (*Tead4*, *Yap1* and *Wwtr1*) (Fig. 5a), and key TFs for myoblast differentiation (*MyoD* and *MyoG*) (Fig. 1b). The correlation analysis results suggested that the two datasets were highly correlated (*R* = 0.94, *p* < 0.01; Fig. 5b). Additionally, the four target genes were downregulated overall during C2C12 differentiation. These genes were also targeted by their respective upregulated miRNAs. These results indicated that the expression of genes in the Hippo signaling pathway was regulated by differentially expressed miRNAs during C2C12 myoblast differentiation.

Furthermore, the interactions between miRNAs and their target genes were verified via the dual-luciferase reporter system. Fragments of putative binding sites or the corresponding mutated binding sites in the 3′ UTRs of the potential target genes *Mob1b*, *Frmd6*, *Ajuba*, *Ppp1cc*, *Ppp2r1b*, *and Snai2* in the network were selected for plasmid construction (Fig. S7). Overexpression of miRNA mimics significantly decreased the relative luciferase activities in the wild-type groups but not in the mutant construct groups compared with the negative control group (Fig. 5c). In other words, these differentially expressed miRNAs could indeed interact with their predicted target genes. In addition, consistent with our prediction, *Ppp2ca* and *Ppp2cb* could be targeted by miR-133a/b^37^. These results provide further evidence that the Hippo signaling pathway is regulated by differentially expressed miRNAs during myoblast differentiation.

### The Hippo signaling pathway was responsible for C2C12 myoblast differentiation

**Fig. 6:**
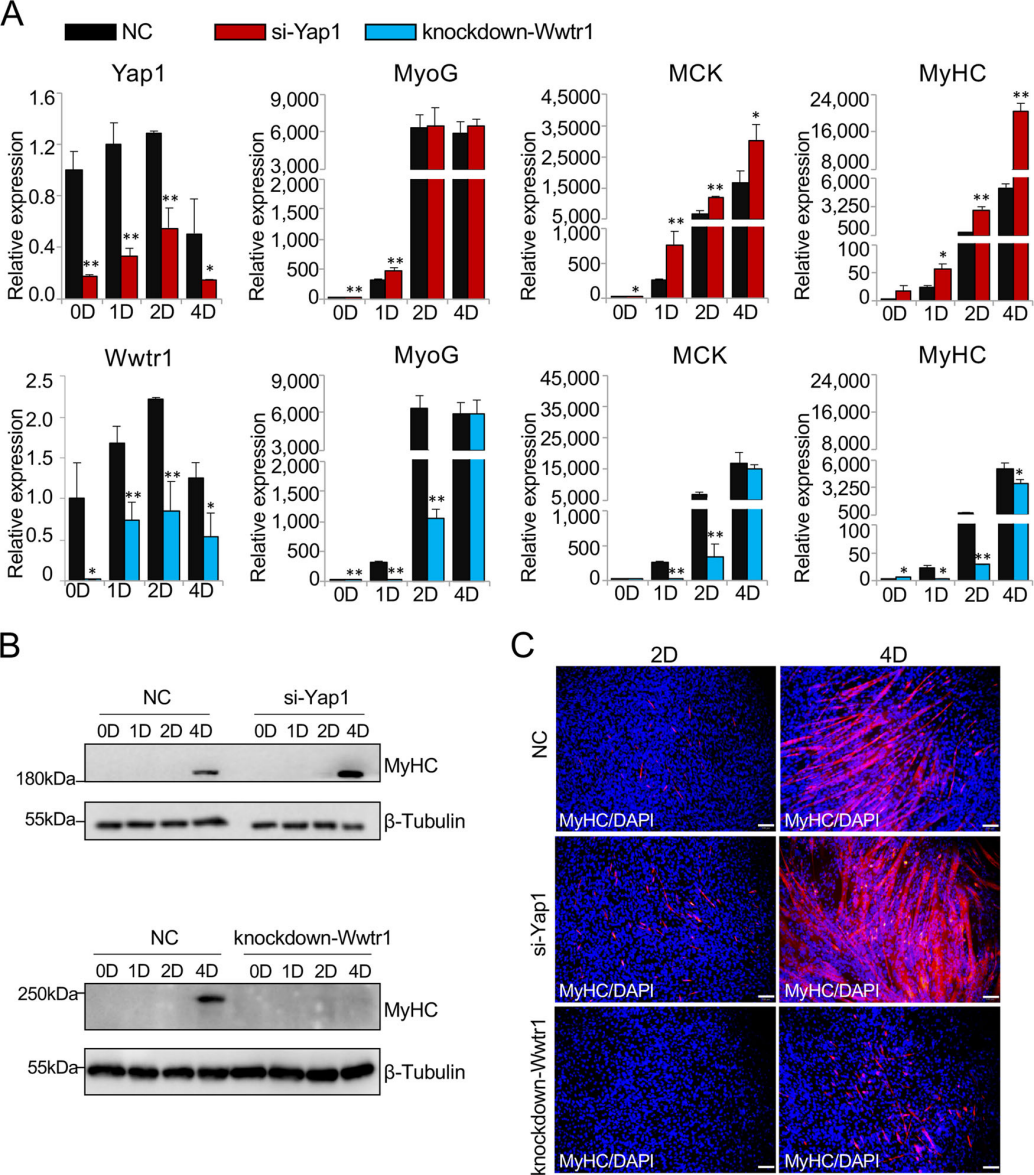
Regulation of myogenic differentiation of C2C12 myoblasts by the Hippo signaling pathway. **a** mRNA expression patterns of genes on days 0, 1, 2, and 4 of cell differentiation via Q-PCR after inhibiting *Yap1* with specific siRNAs (si-Yap) or repressing *Wwtr1* via CRISPR/Cas9 editing (knockdown-Wwtr1) in C2C12 myoblasts. The expression fold change is relative to wild-type C2C12 myoblasts on day 0 of culture in GM (NC). **b**, **c** The protein expression pattern of MyHC during cell differentiation in the si-Yap1, knockdown-Wwtr1, and NC groups as determined via western blotting and immunofluorescence. Myotubes were visualized with an anti-fast myosin antibody (red). Nuclei were stained blue with DAPI. Scale bars: 100 μm. Magnification: ×100. *p < 0.05; **p < 0.01.

To study the effect of the Hippo pathway on C2C12 myoblast differentiation, *Yap1* and *Wwtr1* were significantly inhibited in cells transfected with specific siRNAs against *Yap1* or modified by CRISPR/Cas9 editing for Wwtr1 knockdown (Fig. 6a). The Q-PCR results showed that *MyoG* was significantly upregulated on day 0 and day 1 and that both *MCK* and *MyHC* were significantly upregulated from day 0 to day 4 in the *Yap1* suppression (si-Yap1) group compared to the normal control group (Fig. 6a). However, *MyoG*, *MCK*, and *MyHC* were significantly downregulated when *Wwtr1* was inhibited during cell differentiation (Fig. 6a).

Moreover, as evidenced by western blotting, the protein expression of MyHC was increased on day 4 in the si-Yap1 group but almost disappeared when the expression of *Wwtr1* was inhibited during cell differentiation (Fig. 6b). In addition, the immunofluorescence results revealed that myotube formation was increased when *Yap1* was inhibited but decreased when *Wwtr1* was knocked down during cell differentiation (Fig. 6c). Therefore, we concluded that these two effectors of the Hippo signaling pathway were indeed involved in the regulation of C2C12 myoblast differentiation and played different functional roles; specifically, Yap1 inhibited but Wwtr1 promoted the differentiation process.

### Differentially expressed miRNAs targeted the MAPK signaling pathway during myoblast differentiation

**Fig. 7:**
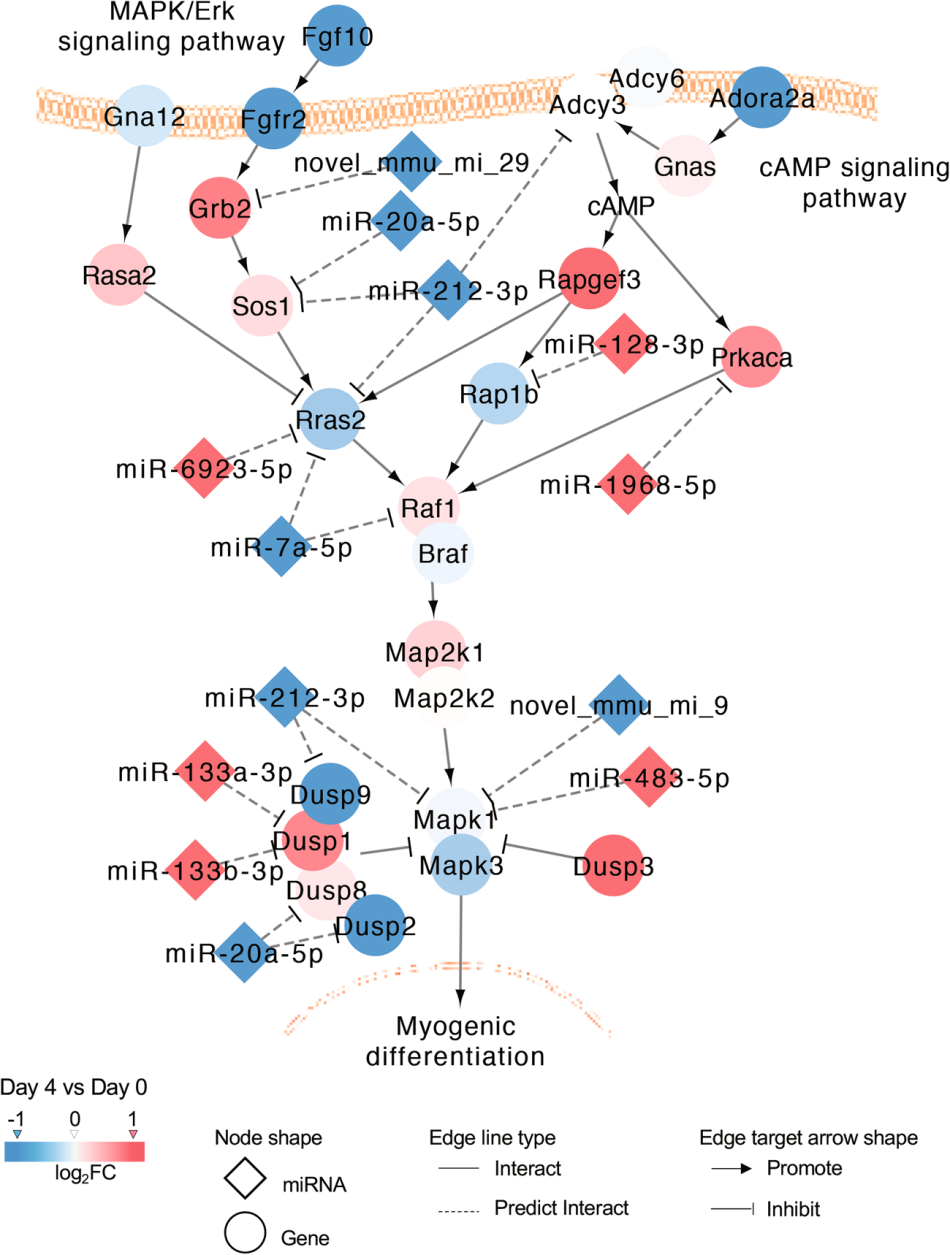
Differentially expressed miRNAs involved in the MAPK signaling pathway and their target genes. Orange red and light red indicate upregulation on day 4 compared with day 0 (orange red, log_2_FC > 1; light red, 0 < log_2_FC < 1), whereas blue and light blue indicate downregulation over this same period (blue, log_2_FC < −1; light blue, −1 < log_2_FC < 0).

Eleven differentially expressed miRNAs were involved in MAPK/Erk signaling pathways, including the cAMP signaling pathway, which has been reported control the regulation of skeletal muscle cell proliferation^38^ and differentiation^39^. The potential network of MAPK signaling pathway-involved differentially expressed miRNAs (miR-128-3p, miR-1968-5p, miR-133a-3p, miR-133b-3p, miR-6923-5p, miR-483-5p, miR-7a-5p, miR-20a-5p, miR-212-3p, novel-mmu-mi-9, and novel-mmu-mi-29) was further investigated using Cytoscape software (Fig. 7, S8, and S9). Moreover, plasmids containing putative binding sites or the corresponding mutated binding sites of the 3′-UTRs of the predicted target genes *Rras2* and *Raf1* (Fig. S10a) were constructed to verify the interactions between these miRNAs and their putative target genes. Predictably, the relative luciferase activities of the wild-type constructs but not the mutant constructs were significantly downregulated after the plasmids were cotransfected with the miRNA mimics (Fig. S10b).

## Discussion

Accumulating studies have shown that miRNAs are involved in many biological processes^8,40,41^, including skeletal myogenesis^10,16^. In this study, we performed a nonbiased analysis to assess miRNA and mRNA expression as well as ATAC-seq and ChIP-seq to investigate chromatin accessibility and TF occupancies, respectively. Changes in chromatin states and MyoD/MyoG binding contributed to the transcriptional regulation of differentially expressed miRNAs. Moreover, these miRNAs regulated muscle differentiation through the Hippo signaling pathway, which was involved in the regulation of MyoD and MyoG and formed feedback regulation loops to promote the differentiation of C2C12 myoblasts.

Nineteen differentially expressed known miRNAs and nine differentially expressed novel miRNAs were identified from miRNA sequencing data collected over the time course of C2C12 myoblast differentiation. These miRNAs included miR-1, miR-133, miR-128, and other miRNAs as shown in the expression patterns identified in previous studies^6,9,14,42^. Combined with the positive results of stem-loop Q-PCR, this evidence further revealed the reliability of our data and analyses. Thus, the novel differentially expressed miRNAs, including three upregulated and six downregulated novel miRNAs, exhibited specific rarely reported expression patterns and might also participate in myogenic differentiation during the time course of C2C12 myoblast differentiation. Most of the differentially expressed miRNAs and their target genes were enriched in pathways involved in muscle proliferation and differentiation, particularly the Hippo signaling pathway.

Hippo family members generally play essential roles in organ size control^43^, tissue regeneration^44^, tumorigenesis^45^, and skeletal muscle development^36^ in mammalian cells. Notably, Yap1 and Wwtr1, downstream effectors of the Hippo signaling pathway, were found to be important regulators during skeletal muscle development^36,46,47^. Inhibition of *Yap1* expression promoted myogenic differentiation in our study, consistent with the results of a previous study^48^. In addition, Yap1-Tead1 directly drove the expression of Snai2 (a known inhibitor of MyoD and Mef2 activities). As another key downstream effector of the Hippo signaling pathway, Taz (encoded by *Wwtr1*) shares overlapping functions with Yap1 in promoting myoblast proliferation^48^. Interestingly, Taz switched to enhance myogenic differentiation by interacting with its cofactor Tead4^48,49^ and with MyoD^50^, as well as via other complex mechanisms^51^. Moreover, we showed that knockdown of *Wwtr1* inhibited myogenic differentiation, indicating that *Wwtr1* indeed had a positive regulatory effect on myoblast differentiation. In our data, most upstream members of the Hippo signaling pathway showed a trend toward downregulation and these genes were negatively regulated by primarily upregulated miRNAs, especially the muscle-specific miRNAs miR-1a-3p, miR-206-3p, and miR-133a/b. Moreover, previous studies showed that the activity of Yap1 and Wwtr1 was negatively regulated by the upstream regulatory elements of the Hippo signaling pathway^36,52^. In summary, the differentially expressed miRNAs altered the abundance of Hippo pathway members. Given that especially active YAP-TEAD signaling promotes myoblast proliferation and inhibits differentiation, it seems likely that differentially expressed miRNAs affect differentiation through the modulation of Hippo signaling.

In addition, 11 differentially expressed miRNAs (miR-128-3p, miR-1968-5p, miR-133a-3p, miR-133b-3p, miR-6923-5p, miR-483-5p, miR-7a-5p, miR-20a-5p, miR-212-3p, novel-mmu-mi-9, and novel-mmu-mi-29) participated in the MAPK/Erk signaling pathway. Unlike members of the Hippo signaling pathway, the upstream signaling molecules contributing to the activity of Mapk1 and Mapk3 (Mapk1/3, also known as Erk2/1) in the network had no clear expression patterns during the time course of C2C12 myoblast differentiation. Previous studies showed that MAPK/Erk pathway inhibition during myoblast differentiation leads to the suppression of cell proliferation^6,38^ and promotion of cell differentiation^6,39,53^. However, other studies reported the opposite conclusion^54,55^. We found that the expression of Mapk1/3 decreased during myoblast differentiation and they were regulated by upregulated miR-483-5p, two downregulated miRNAs (miR-212-3p and novel-mmu-mi-9), and other genes also repressed by these miRNAs. Our results support the idea that the MAPK/Erk pathway plays a negative role in regulating cell differentiation and thus improve our understanding of the regulatory function of the MAPK/Erk signaling pathway in myoblast differentiation.

In this study, we found that the changes in miRNA expression were consistent with the intensity changes of ATAC-seq around these sequences, indicating that these miRNAs were regulated by changes in chromatin states. Previous studies showed that MyoD and MyoG can bind within a region ~10 kb upstream of the miR-1a-1, miR-1a-2, miR-133a-1, and miR-206 pri-miRNAs to mediate their upregulation in differentiated myotubes^26,28^. Here, we combined ATAC-seq, which could map chromatin accessibility at the genome level, with ChIP-seq and RNA-seq to investigate the motif enrichment profiles of TFs in order to reveal the complicated regulatory mechanism of miRNA transcription. Further analysis revealed that 44.25% of the 113 open chromatin regions defined by ATAC-seq peaks exhibited MyoD or MyoG occupancy. Moreover, the majority of these regions were reciprocally occupied by both MyoD and MyoG. The changes in the MyoD intensity between myoblasts and myotubes were almost consistent with those in the ATAC-seq signals. However, MyoG was significantly increased around all differentially expressed miRNAs on day 2.5, indicating that the increase in MyoG binding could either upregulate or downregulate these miRNAs during myoblast differentiation. Evidently, these two factors collaborated with each other to regulate miRNA expression. Moreover, motifs of other TFs were significantly enriched in the non-MyoD/MyoG-occupied, ATAC-seq-defined open chromatin regions around the differentially expressed miRNAs. Furthermore, the expression profiles of these TFs were indeed changed during myoblast differentiation. These results indicated that the TFs in the open chromatin regions can regulate miRNA expression. Our study improved the understanding of the function of chromatin states and the TFs involved in the regulation of miRNA expression during mammalian cell differentiation.

**Fig. 8:**
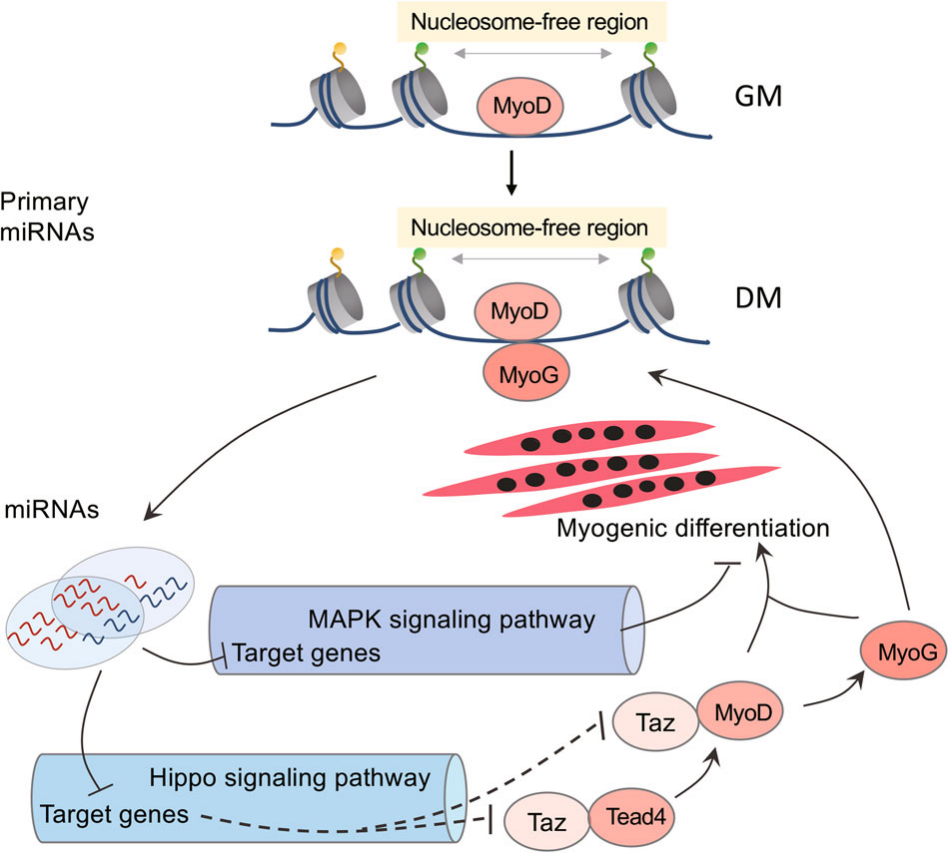
miRNAs and TFs coregulatory network acting through the Hippo and MAPK signaling pathways during C2C12 myoblast differentiation. Chromatin accessibility and TFs occupancies were associated with the expression of miRNAs, miRNAs and TFs formed feedback regulation loops through the Hippo and MAPK signaling pathways to regulate C2C12 myoblast differentiation.

Overall, the two TFs MyoD and MyoG participated in the transcriptional regulation of miRNAs by binding to regions surrounding the pri-miRNAs associated with chromatin accessibility, which in turn regulated muscle differentiation mainly through the Hippo and MAPK signaling pathways. Consequently, feedback regulation loops formed during C2C12 myoblast differentiation (Fig. 8). This study provided new insights into the complex networks underlying the regulation of miRNA expression during skeletal muscle development and the regulation of miRNA biogenesis.

## Supplementary information

Supplementary Figure 1

Supplementary Figure 2

Supplementary Figure 3

Supplementary Figure 4

Supplementary Figure 5

Supplementary Figure 6

Supplementary Figure 7

Supplementary Figure 8

Supplementary Figure 9

Supplementary Figure 10

Supplementary Figure Legends

Supplementary Table 1

Supplementary Table 2

Supplementary Table 3

Supplementary Table 4

Supplementary Table 5

Supplementary Table 6

Supplementary Table Legends

The reproducibility checklist

Declaration of contributions to article

Change of authorship request form - Journals
