# Supplementary material for: Chromatin accessibility is associated with the changed expression of miRNAs that target members of the Hippo pathway during myoblast differentiation
Source: Cell Death Dis. 2020 Feb 24;11(2):148. doi: 10.1038/s41419-020-2341-3 (PMC7039994; doi:10.1038/s41419-020-2341-3)
Supplement: Supplementary file 11 — Supplementary Figure Legends [file 41419_2020_2341_MOESM11_ESM.docx]

**Supplementary Figure 1.** (**A**), (**B**) The protein expression patterns of MyoD and MyoG on days 0, 1, 2, and 4 during C2C12 differentiation by western blotting and immunofluorescence. MyoD and MyoG were indicated as red. Nuclei were stained blue with DAPI. Scale bars: 50 μm. Magnification: 200×.

**Supplementary Figure 2.** GO terms most significantly enriched in the top 20 differentially expressed miRNA targets ranked by the enrichment scores (−log_10_(*p* value)). Node size indicates the number of enriched genes, and color represents the enrichment degree.

**Supplementary Figure 3.** Heat map showing all top 20 KEGG pathways of differentially expressed miRNA targets ranked by the enrichment scores (−log_10_(*p* value)).

**Supplementary Figure 4.** Heat map and clustering patterns of miRNA targets in the MAPK signaling pathway during C2C12 differentiation as determined by rRNA-depleted RNA-seq data.

**Supplementary Figure 5.** Differentially expressed miRNAs involved in the Hippo signaling pathway and their target genes. Orange red and light red indicate upregulation (orange red, log_2_FC > 1; light red, 0 < log_2_FC < 1), whereas blue and light blue indicate downregulation over this same period (blue, log_2_FC < −1; light blue, −1 < log_2_FC < 0).

**Supplementary Figure 6.** Differentially expressed miRNAs involved in the Hippo signaling pathway and their target genes. Orange red and light red indicate upregulation (orange red, log_2_FC > 1; light red, 0 < log_2_FC < 1), whereas blue and light blue indicate downregulation over this same period (blue, log_2_FC < −1; light blue, −1 < log_2_FC < 0).

**S****upplementary Figure 7.** Predicted miRNA binding sites and corresponding mutated binding sites in the 3′-UTRs of target genes.

**Supplementary Figure 8.** Differentially expressed miRNAs involved in the MAPK signaling pathway and their target genes. Orange red and light red indicate upregulation (orange red, log_2_FC > 1; light red, 0 < log_2_FC < 1), whereas blue and light blue indicate downregulation over this same period (blue, log_2_FC < −1; light blue, −1 < log_2_FC < 0).

**Supplementary Figure 9.** Differentially expressed miRNAs involved in the MAPK signaling pathway and their target genes. Orange red and light red indicate upregulation (orange red, log_2_FC > 1; light red, 0 < log_2_FC < 1), whereas blue and light blue indicate downregulation over this same period (blue, log_2_FC < −1; light blue, −1 < log_2_FC < 0).

**Supplementary Figure 10.** Validation of interactions between miRNAs and their putative target genes related to MAPK signaling pathway. (A) Predicted miRNA binding sites and corresponding mutated binding sites in the 3′-UTRs of target genes. **(**B**)** The relative luciferase activities were measured after cotransfection of BHK-21 cells with psiCHECK-2 constructs that contained putative binding sites or the corresponding mutated binding sites in the 3′-UTRs of potential target genes and either miRNA mimics or negative control (NC) for 24 h. **p* < 0.05; ***p* < 0.01.
