# Supplementary material for: Chromatin accessibility is associated with the changed expression of miRNAs that target members of the Hippo pathway during myoblast differentiation
Source: Cell Death Dis. 2020 Feb 24;11(2):148. doi: 10.1038/s41419-020-2341-3 (PMC7039994; doi:10.1038/s41419-020-2341-3)
Supplement: Supplementary file 12 — Supplementary Table 1 [file 41419_2020_2341_MOESM12_ESM.docx]

**Supplementary Table 1. Differentially expressed miRNAs during C2C12 myoblast differentiation.**

| **Names** | **Sequences of mature miRNAs** | **log2FC** | | | | | |
| --- | --- | --- | --- | --- | --- | --- | --- |
|  |  | **1D/0D** | **2D/0D** | **4D/0D** | **2D/1D** | **4D/1D** | **4D/2D** |
| mmu-miR-128-3p_mmu-mir-128-1 | ucacagugaaccggucucuuu | 0.09 | 1.53 | 3.43 | 1.45 | 3.34 | 1.89 |
| mmu-miR-128-3p_mmu-mir-128-2 | ucacagugaaccggucucuuu | 0.03 | 1.37 | 3.08 | 1.34 | 3.05 | 1.71 |
| mmu-miR-133a-3p_mmu-mir-133a-1 | uuugguccccuucaaccagcug | 3.07 | 4.62 | 5.47 | 1.55 | 2.40 | 0.85 |
| mmu-miR-133a-3p_mmu-mir-133a-2 | uuugguccccuucaaccagcug | 3.07 | 4.62 | 5.47 | 1.55 | 2.40 | 0.85 |
| mmu-miR-133a-5p_mmu-mir-133a-1 | gcugguaaaauggaaccaaau | 5.13 | 7.65 | 8.78 | 2.52 | 3.65 | 1.14 |
| mmu-miR-133a-5p_mmu-mir-133a-2 | gcugguaaaauggaaccaaau | 5.13 | 7.62 | 8.79 | 2.49 | 3.66 | 1.17 |
| mmu-miR-133b-3p_mmu-mir-133b | uuugguccccuucaaccagcua | 3.26 | 4.25 | 4.59 | 0.98 | 1.32 | 0.34 |
| mmu-miR-133b-5p_mmu-mir-133b | gcuggucaaacggaaccaaguc | 2.41 | 3.21 | 3.03 | 0.80 | 0.62 | -0.18 |
| mmu-miR-143-5p_mmu-mir-143 | ggugcagugcugcaucucugg | -0.43 | 1.76 | 2.19 | 2.18 | 2.62 | 0.43 |
| mmu-miR-1946a_mmu-mir-1946a | agccgggcagugguggcacacacuuuu | -4.09 | -5.06 | -6.96 | -0.96 | -2.86 | -1.90 |
| mmu-miR-1946b_mmu-mir-1946b | gccgggcagugguggcacaugcuuuu | -3.92 | -4.93 | -3.78 | -1.01 | 0.14 | 1.15 |
| mmu-miR-1968-5p_mmu-mir-1968 | ugcagcuguuaaggaugguggacu | -0.09 | 0.90 | 3.70 | 0.99 | 3.78 | 2.79 |
| mmu-miR-1a-3p_mmu-mir-1a-1 | uggaauguaaagaaguauguau | 4.46 | 7.74 | 9.87 | 3.28 | 5.41 | 2.14 |
| mmu-miR-1a-3p_mmu-mir-1a-2 | uggaauguaaagaaguauguau | 4.46 | 7.74 | 9.87 | 3.28 | 5.41 | 2.14 |
| mmu-miR-206-3p_mmu-mir-206 | uggaauguaaggaagugugugg | 2.39 | 3.84 | 4.58 | 1.45 | 2.19 | 0.74 |
| mmu-miR-20a-5p_mmu-mir-20a | uaaagugcuuauagugcagguag | -2.16 | -3.29 | -3.72 | -1.13 | -1.56 | -0.43 |
| mmu-miR-212-3p_mmu-mir-212 | uaacagucuccagucacggcca | 2.83 | 1.27 | -1.75 | -1.56 | -4.58 | -3.02 |
| mmu-miR-345-3p_mmu-mir-345 | ccugaacuaggggucuggagac | -0.13 | -0.32 | -2.04 | -0.19 | -1.91 | -1.72 |
| mmu-miR-483-5p_mmu-mir-483 | aagacgggagaagagaagggag | 2.09 | 4.37 | 4.84 | 2.28 | 2.75 | 0.47 |
| mmu-miR-499-5p_mmu-mir-499 | uuaagacuugcagugauguuu | 3.30 | 6.41 | 8.59 | 3.11 | 5.29 | 2.18 |
| mmu-miR-6923-5p_mmu-mir-6923 | gugagggcaggaggauuggggugu | 6.21 | 8.35 | 8.61 | 2.14 | 2.40 | 0.27 |
| mmu-miR-7a-5p_mmu-mir-7a-1 | uggaagacuagugauuuuguugu | -1.98 | -3.84 | -4.90 | -1.85 | -2.92 | -1.06 |
| mmu-miR-7a-5p_mmu-mir-7a-2 | uggaagacuagugauuuuguugu | -1.98 | -3.82 | -4.89 | -1.84 | -2.90 | -1.06 |
| mmu-miR-92a-1-5p_mmu-mir-92a-1 | agguugggauuugucgcaaugcu | -3.00 | -2.63 | -4.89 | 0.37 | -1.89 | -2.26 |
| novel_mmu_mi_9 | uugugauucaguggcagagugcu | -0.71 | -11.82 | -11.82 | -11.10 | -11.10 | 0.00 |
| novel_mmu_mi_10 | ccggggugaucggauggccgu | -0.58 | -11.79 | -11.79 | -11.21 | -11.21 | 0.00 |
| novel_mmu_mi_20 | ugcuaugaugaaggcuauguuggua | 11.77 | 11.38 | 0.00 | -0.40 | -11.77 | -11.38 |
| novel_mmu_mi_29 | gggaggggguggggggaaa | -11.63 | -11.63 | -1.24 | 0.00 | 10.40 | 10.40 |
| novel_mmu_mi_36 | ggggguguagcucagugguagag | -0.40 | -6.22 | -15.65 | -5.81 | -15.25 | -9.44 |
| novel_mmu_mi_37 | gaggguuggggggaggcuu | 10.25 | 12.52 | 10.93 | 2.27 | 0.67 | -1.60 |
| novel_mmu_mi_39 | guagggguggggggcguuu | 11.71 | 12.10 | 10.55 | 0.40 | -1.16 | -1.55 |
| novel_mmu_mi_46 | gaccucuugggaucgcgucugga | 3.04 | 0.68 | 0.57 | -2.36 | -2.47 | -0.11 |
| novel_mmu_mi_47 | agaagcuggguugagagggca | -0.66 | -3.71 | -0.05 | -3.06 | 0.61 | 3.67 |
