# Supplementary material for: Chromatin accessibility is associated with the changed expression of miRNAs that target members of the Hippo pathway during myoblast differentiation
Source: Cell Death Dis. 2020 Feb 24;11(2):148. doi: 10.1038/s41419-020-2341-3 (PMC7039994; doi:10.1038/s41419-020-2341-3)
Supplement: Supplementary file 13 — Supplementary Table 2 [file 41419_2020_2341_MOESM13_ESM.docx]

**Supplementary Table 2. Numbers of potential target genes of differentially expressed known miRNAs.**

| **Names of DE known miRNAs** | **Numbers of target genes predicted by TargetScan** | **Numbers of target genes predicted by miRDB** | **Overlap** |
| --- | --- | --- | --- |
| mmu-miR-128-3p | 1097 | 676 | 374 |
| mmu-miR-133a-3p | 616 | 303 | 194 |
| mmu-miR-133a-5p | 3286 | 189 | 181 |
| mmu-miR-133b-3p | 616 | 606 | 194 |
| mmu-miR-133b-5p | 4098 | 225 | 210 |
| mmu-miR-143-5p | 4426 | 242 | 225 |
| mmu-miR-1946a | 1323 | 141 | 134 |
| mmu-miR-1946b | 1343 | 67 | 60 |
| mmu-miR-1968-5p | 5503 | 529 | 510 |
| mmu-miR-1a-3p | 729 | 401 | 267 |
| mmu-miR-206-3p | 729 | 400 | 269 |
| mmu-miR-20a-5p | 1120 | 718 | 557 |
| mmu-miR-212-3p | 414 | 311 | 139 |
| mmu-miR-345-3p | 4667 | 234 | 96 |
| mmu-miR-483-5p | 962 | 45 | 40 |
| mmu-miR-499-5p | 346 | 216 | 88 |
| mmu-miR-6923-5p | 4013 | 313 | 293 |
| mmu-miR-7a-5p | 508 | 434 | 174 |
| mmu-miR-92a-1-5p | 3735 | 221 | 208 |
