# Supplementary material for: Chromatin accessibility is associated with the changed expression of miRNAs that target members of the Hippo pathway during myoblast differentiation
Source: Cell Death Dis. 2020 Feb 24;11(2):148. doi: 10.1038/s41419-020-2341-3 (PMC7039994; doi:10.1038/s41419-020-2341-3)
Supplement: Supplementary file 14 — Supplementary Table 3 [file 41419_2020_2341_MOESM14_ESM.docx]

**Supplementary Table 3. Numbers of potential target genes of differentially expressed novel miRNAs.**

| **Names of DE novel miRNAs** | **numbers of target genes predicted by IntaRNA** | **Numbers of target genes predicted by DIANA** | **Overlap** |
| --- | --- | --- | --- |
| novel_mmu_mi_9 | 1821 | 623 | 27 |
| novel_mmu_mi_10 | 1545 | 79 | 10 |
| novel_mmu_mi_20 | 1334 | 375 | 15 |
| novel_mmu_mi_29 | 1380 | 1232 | 118 |
| novel_mmu_mi_36 | 1924 | 341 | 26 |
| novel_mmu_mi_37 | 1589 | 344 | 16 |
| novel_mmu_mi_39 | 1425 | 139 | 5 |
| novel_mmu_mi_46 | 2137 | 282 | 17 |
| novel_mmu_mi_47 | 1371 | 760 | 38 |
