# Supplementary material for: Chromatin accessibility is associated with the changed expression of miRNAs that target members of the Hippo pathway during myoblast differentiation
Source: Cell Death Dis. 2020 Feb 24;11(2):148. doi: 10.1038/s41419-020-2341-3 (PMC7039994; doi:10.1038/s41419-020-2341-3)
Supplement: Supplementary file 15 — Supplementary Table 4 [file 41419_2020_2341_MOESM15_ESM.docx]

**Supplementary Table 4. cDNA synthesis and Q-PCR primers of miRNAs.**

| **Name** | **Sequence (5'-3')** |
| --- | --- |
| miR-128-3p-RT | CTCAACTGGTGTCGTGGAGTCGGCAATTCAGTTGAGAAAGAGAC |
| miR-1968-5p-RT | CTCAACTGGTGTCGTGGAGTCGGCAATTCAGTTGAGAGTCCACC |
| miR-1a-3p-RT | CTCAACTGGTGTCGTGGAGTCGGCAATTCAGTTGAGATACATAC |
| miR-133b-5p-RT | CTCAACTGGTGTCGTGGAGTCGGCAATTCAGTTGAGGACTTGGT |
| miR-143-5p-RT | CTCAACTGGTGTCGTGGAGTCGGCAATTCAGTTGAGCCAGAGAT |
| miR-7a-5p-RT | CTCAACTGGTGTCGTGGAGTCGGCAATTCAGTTGAGACAACAAA |
| miR-128-3p-F | TCGGCAGGTCACAGTGAACCGGT |
| miR-1968-5p-F | TCGGCAGGTGCAGCTGTTAAGGA |
| miR-1a-3p-F | TCGGCAGGTGGAATGTAAAGAAG |
| miR-133b-5p-F | TCGGCAGGGCTGGTCAAACGGA |
| miR-143-5p-F | TCGGCAGGGGTGCAGTGCTGC |
| miR-7a-5p-F | TCGGCAGGTGGAAGACTAGTGAT |
| U6-F | GTGCTCGCTTCGGCAGCACATAT |
| U6-R | AAAATATGGAACGCTTCACGAA |
| universal primer-R | TCAACTGGTGTCGTGGAGTCGGC |
