# Supplementary material for: Chromatin accessibility is associated with the changed expression of miRNAs that target members of the Hippo pathway during myoblast differentiation
Source: Cell Death Dis. 2020 Feb 24;11(2):148. doi: 10.1038/s41419-020-2341-3 (PMC7039994; doi:10.1038/s41419-020-2341-3)
Supplement: Supplementary file 16 — Supplementary Table 5 [file 41419_2020_2341_MOESM16_ESM.docx]

**Supplementary Table 5. Q-PCR primers of mRNAs.**

| **Name** | **Sequence (5'-3')** |
| --- | --- |
| MyoD-F | GCAGGCTCTGCTGCGCGACC |
| MyoD-R | TGCAGTCGATCTCTCAAAGCACC |
| MyoG-F | CAATGCACTGGAGTTCGGT |
| MyoG-R | CTGGGAAGGCAACAGACAT |
| MCK-F | GCTTATGGTGGAGATGGAGA |
| MCK-R | GGCCATCACGGACTTTTATT |
| MyHC2d-F | GGACCCACGGTCGAAGTTG |
| MyHC2d-R | CCCGAAAACGGCCATCT |
| Rap1b-F | TGTGTGTACAGTGGGGGTCT |
| Rap1b-R | ACCGAAACGTTACACATGGGA |
| Rras2 -F | AGTTTGGAGCCTGAGCTGTT |
| Rras2 -R | GCCGTAGCATCAGGAAAGGT |
| Spp1-F | GTTCTCCTGGCTGAATTCTGAGGG |
| Spp1-R | CATGTGGCTATAGGATCTGGGTG |
| Ndufs2-F | CCAGGAGCCACATATACTGCC |
| Ndufs2-R | TGCTTTCGCTCATCGGTCTAT |
| Adora1-F | GGCCACAGACCTACTTCCAC |
| Adora1-R | CGCTGAGTCACCACTGTCTT |
| Ajuba-F | CCCTGGTTGCTGTTTTGCTC |
| Ajuba-R | GTGGTGGGGTTGTAACAGGT |
| Ywhab-F | CGCTGCCCTCTGATCTTCCC |
| Ywhab-R | TTCGATGCTGGAGATGACACG |
| Stk4-F | GGTGGCTTCTGGGTTGCTA |
| Stk4-R | CTGCTCCTGGGGTCTAGAAT |
| Ppp1cc-F | CCCAACTACTGTGGCGAGTTT |
| Ppp1cc-R | GTGGACGGCAAGTTAGTTCCTT |
| Tead4-F | ACTCAAGTTTTGGCAAGGAGC |
| Tead4-R | AGCCGAGAACTCCAACATCC |
| Yap1-F | CCCTCGTTTTGCCATGAACC |
| Yap1-R | TCCGTATTGCCTGCCGAAAT |
| Wwtr1-F | CCTCAGCAACATGGACGAGA |
| Wwtr1-R | TCATCACTTGGTCCGCAAGG |
| GAPDH-F | CACGGCAAATTCAACGGCACAGTCAAGG |
| GAPDH-R | GTTCACACCCATCACAAACATGG |
