# Supplementary material for: Chromatin accessibility is associated with the changed expression of miRNAs that target members of the Hippo pathway during myoblast differentiation
Source: Cell Death Dis. 2020 Feb 24;11(2):148. doi: 10.1038/s41419-020-2341-3 (PMC7039994; doi:10.1038/s41419-020-2341-3)
Supplement: Supplementary file 17 — Supplementary Table 6 [file 41419_2020_2341_MOESM17_ESM.docx]

**Supplementary Table 6. Luciferase activity assay primers of 3′ UTR fragments containing predicted miRNA binding sites of mRNAs.**

| **Name** | **Sequence (5'-3')** |
| --- | --- |
| mob1b 3‘UTR-F | CCGCTCGAGTCAACAGCCGTTTAAAGTTCTAC |
| mob1b 3‘UTR-R | ATAAGAATGCGGCCGCCCGTTACAGGGGTGACAGAAA |
| Ajuba 3‘UTR-F | CCGCTCGAGGCGGAGGCAAGGAGTGTATT |
| Ajuba 3‘UTR-R | ATAAGAATGCGGCCGCACACGGGGAGCTCTCTAGTC |
| Snai2 3‘UTR-F | CCGCTCGAGCACTGCTGCCAAACCATTTCA |
| Snai2 3‘UTR-R | ATAAGAATGCGGCCGCGCACTTGGAGGGGCATTGTA |
| Rras2 3‘UTR-F | CCGCTCGAGGGAGCCTGAGCTGTTTGCTA |
| Rras2 3‘UTR-R | ATAAGAATGCGGCCGCACAGCTTTAGCTTGTGTGGGT |
| Raf1 3‘UTR-F | CCGCTCGAGAGCAGGCACCACTTTCTGTT |
| Raf1 3‘UTR-R | ATAAGAATGCGGCCGCCCTGGCTTTCTTACAGACAAGGA |
