# Supplementary material for: Chromatin accessibility is associated with the changed expression of miRNAs that target members of the Hippo pathway during myoblast differentiation
Source: Cell Death Dis. 2020 Feb 24;11(2):148. doi: 10.1038/s41419-020-2341-3 (PMC7039994; doi:10.1038/s41419-020-2341-3)
Supplement: Supplementary file 18 — Supplementary Table Legends [file 41419_2020_2341_MOESM18_ESM.docx]

**Supplementary Table 1.** Differentially expressed miRNAs during C2C12 myoblast differentiation.

**Supplementary Table 2.** Numbers of potential target genes of differentially expressed known miRNAs.

**Supplementary Table 3.** Numbers of potential target genes of differentially expressed novel miRNAs.

**Supplementary Table 4.** cDNA synthesis and Q-PCR primers of miRNAs.

**Supplementary Table 5.** Q-PCR primers of mRNAs.

**Supplementary Table 6.** Luciferase activity assay primers of 3′ UTR fragments containing predicted miRNA binding sites of mRNAs.
