# Supplementary material for: Chromatin accessibility is associated with the changed expression of miRNAs that target members of the Hippo pathway during myoblast differentiation
Source: Cell Death Dis. 2020 Feb 24;11(2):148. doi: 10.1038/s41419-020-2341-3 (PMC7039994; doi:10.1038/s41419-020-2341-3)
Supplement: Supplementary file 20 — Declaration of contributions to article [file 41419_2020_2341_MOESM20_ESM.pdf]

DECLARATION OF CONTRIBUTIONS TO ARTICLE

ADMC

Manuscript Number:	Journal Name:	
CDDIS-19-2092R	*Cell Death & Disease*	(the 'Journal')

Proposed Title of the Contribution:

MiRNAs targeting the Hippo signaling pathway during myoblast differentiation are regulated by changes of chromatin states	(the 'Contribution')

Author(s):

Huanhuan Zhou, Yue Xiang, Mingyang Hu, Yueyuan Xu, Ye Hou, Xiaolong Qi, Liangliang Fu, Yu Luan, Zhangxu Wang, Xinyun Li, Yunxia Zhao and Shuhong Zhao	(the 'Authors')

For all *CDDis* articles, each person named as an author in the published version must be able to show he or she has contributed substantially to the article.

Authorship credit should be based on 1) substantial contributions to conception and design, acquisition of data, or analysis and interpretation of data; 2) drafting the article or revising it critically for important intellectual content; and 3) final approval of the version to be published. Authors should meet conditions 1, 2 and 3.

Any person who cannot be shown to have made a substantial contribution to the article cannot be listed as an author in the final version. The name of any person who is deemed to have made a minor contribution can, however, appear in the Acknowledgments section of the article.

Please complete the table below to indicate the contributions of all named authors to the manuscript.

Author Full Name:	Specification of Contribution to the Manuscript:
Huanhuan Zhou	H.Z. conceived and performed the experiments and analyzed a part of data and explained the data. She also drafted, wrote and revised the manuscript and approved the version to be published.
Yue Xiang	Y.X. analyzed and helped explain the data. She also helped draft and revised the manuscript and approved the version to be published.
Mingyang Hu	M.H. analyzed and helped explain the data. He also helped draft and revised the manuscript and approved the version to be published.
Yueyuan Xu	Y.Y.X. analyzed and helped explain the data. She also helped draft and revised the manuscript and approved the version to be published.
Ye Hou	Y.H. assisted in the experiments and helped explain the data. She also helped draft and revised the manuscript and approved the version to be published.
Xiaolong Qi	X.Q. assisted in the experiments and helped explain the data. He also helped draft and revised the manuscript and approved the version to be published.
Liangliang Fu	L.F. helped conceive the experiments and explained the data. He also revised the manuscript and approved the version to be published.
Yu Luan	Y.L. helped conceive the experiments and explained the data. He also revised the manuscript and approved the version to be published.
Zhangxu Wang	Z.W. assisted in the experiments and explained the data. He also revised the manuscript and approved the version to be published.
Xinyun Li	X.L. helped conceive the experiments and explained the results. He also revised it critically for important intellectual content and approved the version to be published.
Yunxia Zhao	Y.Z. designed the study and explained the data . She also revised it critically for important intellectual content and approved the version to be published
Shuhong Zhao	S.Z. designed the study and explained the results . She also revised it critically for important intellectual content and approved the version to be published.

Please complete the table below to indicate the contributions of all named authors to the figures.

Figure 1:

H.Z. cultured C2C12 cells (Fig. 1A), generated the Q-PCR data, performed the correlation analysis (Fig. 1B, 1D, and 1E), and labelled and assembled all figures.
Y.X. analyzed the miRNA-seq data and generated the heatmap (Fig. 1C).
Y.H. and Z.W. assisted in the Q-PCR experiments (Fig. 1B, 1D, and 1E).

Figure 2:

X.Q. generated and Y.Y.X. analyzed the ATAC-seq data (Fig. 2A).
Y.Y.X. analyzed the Chip-seq data with the assistance of Y.L. (Fig. 2).
M.H. analyzed the data and generated corresponding figures with the assistance of Y.Z. and Y.L. (Fig. 2B-2K).
H.Z. generated figure 2A and 2H, labelled and assembled all figures.

Figure 3:

H.Z. analyzed the data of targets of known miRNA.
Y.X. analyzed the data of targets of novel miRNA and generated the figures (Fig. 3A and 3B).
H.Z. generated the Q-PCR assay (Fig. 3C), performed the correlation analysis (Fig. 3D), and labelled and assembled all figures.

Figure 4:

H.Z. generated the network and assembled the figure (Fig. 4) with the assistance of Y.H..

Figure 5:

H.Z. performed the Q-PCR assay (Fig. 5A) and the correlation analysis (Fig. 5B) with the assistance of Y.H. and Z.W..
H.Z. performed the dual luciferase report assay with the assistance of L.F. (Fig. 5C), labelled and assembled all figures.

Figure 6:

H.Z. performed the RNAi assay and generated the knockdown cell with the assistance of L.F..
H.Z. carried out the Q-PCR (Fig. 6A), western blotting (Fig. 6B) and immunofluorescence assay (Fig. 6C) with the assistance of L.F..
H.Z. also labelled and assembled all figures.H.Z. generated the network and labelled and assembled all figures (Fig. 7 and 8).
Y.L. helped generate the network (Fig.8).
In addition, all author helped revise the manuscript and approved the version to be published.

Signed for and on behalf of the Author(s):	Print Name:	Date:
Yunxia Zhao Shuhong Zhao	**Yunxia Zhao** **Shuhong Zhao**	11-20-2019
