# Supplementary material for: Chromatin accessibility is associated with the changed expression of miRNAs that target members of the Hippo pathway during myoblast differentiation
Source: Cell Death Dis. 2020 Feb 24;11(2):148. doi: 10.1038/s41419-020-2341-3 (PMC7039994; doi:10.1038/s41419-020-2341-3)
Supplement: Supplementary file 21 — Change of authorship request form - Journals [file 41419_2020_2341_MOESM21_ESM.pdf]

SPRINGER NATURE

Change of authorship request form - Journals (pre-acceptance)

Important information. Please read.

- This form should be used by authors to request any change in authorship (adding/deleting authors) including changes in corresponding authors. This form should not be used for name changes. Please fully complete all sections. Use black ink and block capitals and provide each author's full name with the given name first followed by the family name.

- By signing this declaration, all authors guarantee that the order of the authors are in accordance with their scientific contribution, if applicable as different conventions apply per discipline, and that only authors have been added who made a meaningful contribution to the work.

- Please note, in author collaborations where there is formal agreement for representing the collaboration, it is sufficient for the representative or legal guarantor (usually the corresponding author) to complete and sign the Authorship Change Form on behalf of all authors, **next to the added/removed author(s). (Complete Section 3, followed by Section 6.)**
  In author collaborations where there is no formal agreement for representing the collaboration and **there are more than 10 authors**, one may sign for all, provided the signer appends correspondence that attests that each of the authors have agreed to the change **and the added/removed authors sign the form**. **(Complete Section 3, followed by Section 6.)**

- Please note, we cannot investigate or mediate any authorship disputes. If you are unable to obtain agreement from all authors (including those who you wish to be removed) you must refer the matter to your institution(s) for investigation. Please inform us if you need to do this.

- If you are not able to return a fully completed form within **30 days** of the date that it was sent to the author requesting the change, we may have to withdraw your manuscript. We cannot publish manuscripts where authorship has not been agreed by all authors (including those who have been removed).

- Incomplete forms will be rejected.

- Please return/upload this form, fully completed, to the Journals Editorial Office. The Journal and/or Publisher will consider the information you have provided to decide whether to approve the proposed change in authorship. We may decide to contact your institution for more information or undertake a further investigation, if appropriate, before making a final decision.

SPRINGER NATURE # Change of authorship request form - Journals (pre-acceptance)

Section 1: Please provide the current title of manuscript

Manuscript ID no.:	CDDIS-19-2092R

Title:	MIRNAS TARGETING THE HIPPO SIGNALING PATHWAY DURING MYOBLAST DIFFERENTIATION ARE REGULATED BY CHANGES OF CHROMATIN STATES

Section 2: Please provide the previous authorship, in the order shown on the manuscript before the changes were introduced. Please indicate the corresponding author by adding (CA) behind the name.

	First name(s)	Family name	ORCID or SCOPUS id, if available
1st author	HUANHUAN	ZHOU	
2nd author	YUE	XIANG	
3rd author	MINGYANG	HU	
4th author	YUEYUAN	XU	
5th author	YE	HOU	
6th author	XIAOLONG	QI	
7th author	YU	LUAN	
8th author	ZHANGXU	WANG	
9th author	XINYUN	LI	
10th author	YUNXIA	ZHAO (CA)	

Please use an additional sheet if there are more than 10 authors.

11th author	SHUHONG	ZHAO (CA)	

SPRINGER NATURE Springer Nature is one of the world's leading global research, educational and professional publishers, created in May 2015 through the combination of Nature Publishing Group, Palgrave Macmillan, Macmillan Education and Springer Science+Business Media.

SPRINGER NATURE

Change of authorship request form - Journals (pre-acceptance)

Section 3: Please provide a justification for change. Please use this section to explain your reasons for changing the authorship of your manuscript, e.g. what necessitated the change in authorship? Please refer to the (journal) policy pages for more information about authorship. Please explain why omitted authors were not originally included and/or why authors were removed on the submitted manuscript.

ACCORDING TO THE REVIEWERS' SUGGESTIONS, WE ADDED THE LOSS-OF-FUNCTION ASSAY FOR HIPPO SIGNALING PATHWAY, THE DUAL LUCIFERASE ASSAY FOR MUTANT BINDING SITES OF MIRNA TARGET GENES, AND WESTERN BLOTTING AND IMMUNOFLUORESCENCE FOR MYOD AND MYOG. FOR THE ABOVE EXPERIMENTS, LIANGLIANG FU NOT ONLY HELPED CONCEIVE THE EXPERIMENTS, BUT ALSO HELPED EXPLAIN THE RESULTS OF THE EXPERIMENTS. HE ALSO REVISED THE MANUSCRIPT AND APPROVED THE VERSION TO BE PUBLISHED. IN VIEW OF THE IMPORTANT CONTRIBUTION HE HAS MADE TO THE REVISED MANUSCRIPT, WE ADD LIANGLIANG FU TO THE AUTHORSHIP LIST.

Section 4: Proposed new authorship. Please provide your new authorship list in the order you would like it to appear on the manuscript. Please indicate the corresponding author by adding (CA) behind the name. If the Corresponding Author has changed, please indicate the reason under section 3.

	First name(s)	Family name (this name will appear in full on the final publication and will be searchable in various abstract and indexing databases)	Affiliated institute	E-mail address
1st author	HUANHUAN	ZHOU	Huazhong Agricultural University	zhouhuanhuanst@163.com
2nd author	YUE	XIANG	Huazhong Agricultural University	yxiang@webmail.hzau.edu.cn
3rd author	MINGYANG	HU	Huazhong Agricultural University	myhu@webmail.hzau.edu.cn
4th author	YUEYUAN	XU	Huazhong Agricultural University	yyxu@webmail.hzau.edu.cn
5th author	YE	HOU	Huazhong Agricultural University	houye2009@126.com
6th author	XIAOLONG	QI	Huazhong Agricultural University	xlqihzau@163.com
7th author	LIANGLIANG	FU	Huazhong Agricultural University	fuliangliang2011@163.com
8th author	YU	LUAN	Huazhong Agricultural University	ly178574378@hotmail.com
9th author	ZHANGXU	WANG	Huazhong Agricultural University	zxwanglwh@163.com
10th author	XINYUN	LI	Huazhong Agricultural University	xyli@mail.hzau.edu.cn

Please use an additional sheet if there are more than 10 authors.

11th author	YUNXIA	ZHAO (CA)	Huazhong Agricultural University	yxzhao@mail.hzau.edu.cn
12th author	SHUHONG	ZHAO (CA)	Huazhong Agricultural University	shzhao@mail.hzau.edu.cn

---
SPRINGER NATURE Springer Nature is one of the world's leading global research, educational and professional publishers, created in May 2015 through the combination of Nature Publishing Group, Palgrave Macmillan, Macmillan Education and Springer Science+Business Media.

SPRINGER NATURE **Change of authorship request form - Journals** (pre-acceptance)

Section 5: Author contribution, Acknowledgement and Disclosures. Please use this section to provide a new disclosure statement and, if appropriate, acknowledge any contributors who have been removed as authors and ensure you state what contribution any new authors made (if applicable per the journal or book (series) policy). Please ensure these are updated in your manuscript - after approval of the change(s) - as our production department will not transfer the information in this form to your manuscript.

New acknowledgements:

New Disclosures (financial and non-financial interests, funding):

New Author Contributions statement (if applicable per the journal policy):

L.F. HELPED CONCEIVE THE EXPERIMENTS AND EXPLAINED THE DATA. HE ALSO REVISED THE MANUSCRIPT AND APPROVED THE VERSION TO BE PUBLISHED.

State 'Not applicable' if there are no new authors.

SPRINGER NATURE

Change of authorship request form - Journals (pre-acceptance)

Section 6: Declaration of agreement. *All* authors, unchanged, new and removed *must* sign this declaration.
(NB: Please print the form, (docu)-sign and return/upload a scanned copy. Please note that signatures that have been inserted as an image file are acceptable as long as it is handwritten. Typed names in the signature box are unacceptable.) *** Please delete as appropriate. Delete all of the bold if you were on the original authorship list and are remaining as an author.**

	First name	Family name		Signature	Date
1st author	HUANHUAN	ZHOU	I agree to the proposed new authorship shown in section 4 and the proposed change in corresponding author	Huanhuan Zhou	11-19-2019
2nd author	YUE	XIANG	I agree to the proposed new authorship shown in section 4 and the proposed change in corresponding author	Yue Xiang	11-19-2019
3rd author	MINGYANG	HU	I agree to the proposed new authorship shown in section 4 and the proposed change in corresponding author	Mingyang Hu	11-19-2019
4th authors	YUEYUAN	XU	I agree to the proposed new authorship shown in section 4 and the proposed change in corresponding author	Yueyuan Xu	11-19-2019
5th author	YE	HOU	I agree to the proposed new authorship shown in section 4 and the proposed change in corresponding author	Ye Hou	11-19-2019
6th author	XIAOLONG	QI	I agree to the proposed new authorship shown in section 4 and the proposed change in corresponding author	Xiaolong Qi	11-19-2019
7th author	LIANGLIANG	FU	I agree to the proposed new authorship shown in section 4 /**and the addition of my name to the authorship list** /and the proposed change in corresponding author	Liangliang Fu	11-19-2019

SPRINGER NATURE Springer Nature is one of the world's leading global research, educational and professional publishers, created in May 2015 through the combination of Nature Publishing Group, Palgrave Macmillan, Macmillan Education and Springer Science+Business Media.

SPRINGER NATURE

Change of authorship request form - Journals (pre-acceptance)

	First name	Family name		Signature	Date
8th author	YU	LUAN	I agree to the proposed new authorship shown in section 4 and the proposed change in corresponding author	Y Luan	11.19.2019
9th author	ZHANGXU	WANG	I agree to the proposed new authorship shown in section 4 and the proposed change in corresponding author	zhangxu wang	11.19.2019
10th author	XINYUN	LI	I agree to the proposed new authorship shown in section 4 and the proposed change in corresponding author	Xinyun Li	11.19.2019

Please use an additional sheet if there are more than 10 authors.

11th author	YUNXIA	ZHAO (CA)	I agree to the proposed new authorship shown in section 4 and the proposed change in corresponding author	Yunxia zhao	11-19-2019
12th author	SHUHONG	ZHAO (CA)	I agree to the proposed new authorship shown in section 4 and the proposed change in corresponding author	Shuhong Zhao	11-20-2019

In case of author collaborations with formal agreement:

	Name of consortium/consortia	First name	Family name		Signature	Date
Representative/legal guarantor				I agree to the proposed new authorship shown in section 4 **/and the addition/removal*of my name to the authorship list** /and the proposed change in corresponding author		

Both added/removed authors should complete the information in the first table under Section 6.

---- End of form ----

SPRINGER NATURE Springer Nature is one of the world's leading global research, educational and professional publishers, created in May 2015 through the combination of Nature Publishing Group, Palgrave Macmillan, Macmillan Education and Springer Science+Business Media.
